# Parametrized statistical appearance and shape modelling strategy to predict proximal and diaphyseal femoral fractures

**DOI:** 10.3389/fbioe.2025.1693678

**Published:** 2025-11-03

**Authors:** Özgür Cebeci, Duane S. Cronin, Sara Checa

**Affiliations:** ^1^ IAT Ingenieurgesellschaft für Automobiltechnik mbH, Berlin, Germany; ^2^ Berlin Institute of Health at Charité - Universitätsmedizin Berlin, Julius Wolff Institute, Berlin, Germany; ^3^ Department of Mechanical and Mechatronics Engineering, University of Waterloo, Waterloo, ON, Canada; ^4^ Institute of Biomechanics, Hamburg University of Technology, Hamburg, Germany

**Keywords:** statistical shape modelling, statistical appearance modelling, parametric femur modelling, finite element model, anthropometric variations, femoral fracture load, fracture prediction

## Abstract

**Introduction:**

Femoral loading leading to a fracture is known to vary with anthropometry, and patient-specific finite element models have provided important insights into fracture prediction but are often very time consuming to generate. Additionally, existing parametric models do not simultaneously account for variations in both femur geometry and bone density distribution and remain limited to either the femoral shaft or the proximal femur. This inhibits their ability to predict fractures involving both the shaft and proximal regions.

**Methods:**

In the present study, a novel parametric femur modeling strategy was developed to create whole femur models based on stature, BMI, and age input, including density distribution and geometrical variations, for fracture loading predictions. A statistical shape and appearance femur model was developed based on an input set of CT scans of healthy female femurs (N = 18) between the ages of 50 and 70. Thereafter, multilinear regressions were used to relate principal components to the subject anthropometric characteristics and develop parametric models. The developed parametric models were evaluated using traditional patient-specific models for their potential to represent the influence of changing patient stature, BMI, and age on femoral fractures. Femoral fracture load in three-point bending, axial torsion, and lateral fall cases was predicted using the parametric as well as subject-specific femur models.

**Results:**

The developed parametric model was able to predict femoral fracture load variations due to changing anthropometry and age with an average difference of 4.85% compared with predictions using subject-specific models.

**Discussion:**

Therefore, this novel parametric femur model can predict fracture loading while directly incorporating the influence of changing patient anthropometry. In the future, the model could support the development of orthopedic devices tailored to specific patient anthropometries to help mitigate femoral fractures.

## 1 Introduction

Proximal femur and femur shaft fractures pose a growing problem due to the increasing life expectancy and the reduced bone quality in the elderly (Veronese and Maggi, 2018). These fractures are related to excessive femur neck bending and bending or torsional loading of the femur shaft (DeGoede et al., 2003; Gitajn and Rodriguez, 2011), often due to falling or stumbling. The mortality rate for the elderly in the first year after a femoral fracture have been reported to be higher than 20% (Lundin et al., 2021).

In order to mitigate femoral fractures, femoral fracture load estimations play a crucial role in identifying fracture risk (Karlamangla et al., 2004). Epidemiological studies have shown that females above the age of 50 have an increased femoral fracture risk (Bergh et al., 2020; Kanis et al., 2013). However, identifying patient groups at higher risk of a femoral fracture and developing customized solutions to avoid fractures remains challenging. Currently, clinical evaluation of the fracture risk relies on dual-energy x-ray absorptiometry (DXA) measurements (IAEA, 2010), which can detect patients with reduced bone quality based on the areal bone mineral density (aBMD) values. Nevertheless, the femur geometry and the three-dimensional bone density distribution are not represented in DXA measurements, which poses a substantial limitation in femoral fracture load predictions (Beck, 2007).

Subject-specific finite element (FE) models, generated based on quantitative computer tomography (QCT) measurements, have been shown to predict subject-specific femoral fracture load with better accuracy (Keaveny et al., 2020; Falcinelli and Whyne, 2020) than clinical aBMD measurements (Blake and Fogelman, 2007). In the last decade using FE analysis, studies also reported accurate prediction of femoral fracture loading and patterns for femoral shaft (Khor et al., 2018) or proximal femur (Enns-Bray et al., 2018) separately. However, subject-specific FE models have the drawbacks of substantial model preparation cost, required QCT measurements, and corresponding radiation exposure (Viceconti et al., 2018).

Accordingly, statistical modeling techniques have recently gained popularity since they provide possibilities to generate personalized femur models with reduced cost and radiation exposure (Sarkalkan et al., 2014; Nolte and Bull, 2019; Grassi et al., 2021). The core of statistical femur modeling is principal component analysis, in which the normalized data set is decomposed to its orthogonal (principal) components, where each component represents a distinct variation in bone shape or density distribution. As a result of the principal component analysis, a subject-specific femur can be represented and reconstructed realistically in terms of the principal values (Grassi et al., 2014). Statistical femur models also enable the generation of parametric femurs, which represent the shape and density variability of a reference set (Bonaretti et al., 2014). These models can be adjusted using a set of parameters, allowing for the generation of realistic femurs based on specific characteristics.

Patient anthropometry influences the loading conditions, which might lead to a femoral fracture (Majumder et al., 2013). Therefore, it has been suggested that alongside age, anthropometry also plays a key role in femoral fracture risk (Palanca et al., 2021; Luo, 2021). Regarding the influence of changing patient anthropometry on bone morphology, previous studies have shown correlations between bone size and stature (Menéndez Garmendia et al., 2018), femoral neck-shaft angle (Fischer et al., 2020) or cortical bone thickness (Thompson, 1980) and age. Accordingly, studies have also shown femoral fracture load variations due to changing age and anthropometry (Shen et al., 2015; Keaveny et al., 2009; Mather, 1968; Funk et al., 2004).

In order to investigate the influence of the changing anthropometry in femoral fractures, based on the required level of significance (Hulley et al., 2013), a large number of patient-specific FE models need to be included to provide statistically meaningful outcomes (Yang et al., 2014; Kopperdahl et al., 2014). Alternatively, a femur model parametrized based on input values like stature, BMI, and age can predict the effect of anthropometric variations on density distribution and femur geometry. Such a model would enable testing fracture mitigation strategies deterministically incorporating the influence of changing anthropometry with a reduced number of simulations.

Klein et al. (2015) using linear regressions developed a parametric shape model that predicted the femur shape as a function of age, stature, and BMI. Although the model was able to predict force displacement behavior under combined compression and bending loading, the models presented several limitations. Among them, homogeneous femur material properties were calibrated to match the average response of the reference experiments (Ivarsson et al., 2009), thereby limiting the potential for future clinical applications.

In order to increase the applicability as a research and design tool, a comprehensive parametric femur model should be able to predict femoral fracture load in fall-induced loading conditions, as well as under femur shaft bending and torsion, since these are common femoral fractures among the elderly (Salminen et al., 2000; DeGoede et al., 2003; Gitajn and Rodriguez, 2011). None of the previous studies so far has attempted to develop a parametric femur model of the whole femur that simultaneously incorporates stature, BMI, and age to predict fracture loading across all these load cases, while accounting for both shape and density distribution variations. Such a holistic model can inform the investigation of fracture risk due to anthropometric variation in complex load cases, such as road accidents or periprosthetic fractures, where the femoral loading cannot be confined to the proximal or shaft region, or simplified to a single loading mode such as bending or torsion.

The aim of this study was to develop and evaluate a novel parametric femur model that integrates stature, BMI, and age as input variables, while simultaneously accounting for both the geometry and density distribution of the whole femur, to predict fracture loading under lateral fall, axial torsion, and shaft bending conditions. The developed parametric model was evaluated in terms of the effect of anthropometric variation on fracture load predictions. The results showed that the developed parametric model can capture the influence of anthropometric variation on femoral fracture loading with good agreement to subject-specific models.

## 2 Methods

**FIGURE 1:**
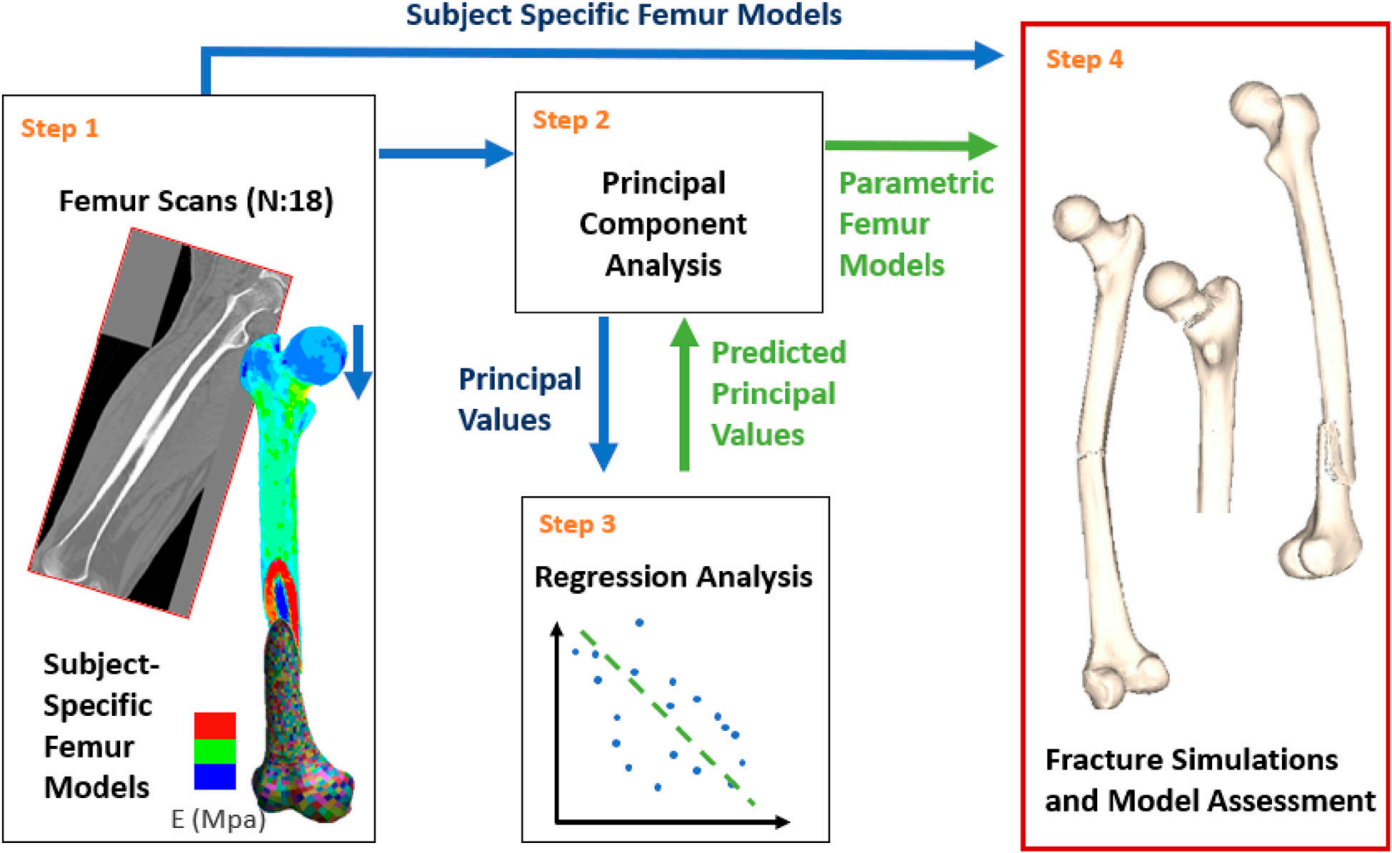
Study overview: Subject-specific models were generated based on the CT scans (Step 1). Principal component analysis was conducted using the subject-specific femurs (Step 2). Multilinear regressions were developed for model parametrization based on the principal component analysis results and the patient anthropometry (Step 3). Finally, subject-specific and parametric femur models were compared under various fracture loading scenarios for model assessment (Step 4). Blue and green arrows represent processes related to subject-specific and parametric femur models, respectively.

A parametric femur model was constructed based on a dataset of 18 femurs. An overview of the workflow (Figure 1), explained in detail below, is as follows:

Step 1. Create subject-specific femur models from individual computer tomography (CT) scan data.
Step 2. Conduct a principal component analysis using the input femur models to identify principal values and components for geometry and the bone density distribution.
Step 3. Perform regression analysis for stature, BMI, age and the principal values associated with the density distribution and geometry.
Step 4. Assessment of the parametric femurs against subject-specific models in terms of the femoral geometry and density distribution alongside the changes in femoral fracture load associated with stature, BMI, and age.

In this study, femoral fracture load was determined using explicit FE models. All simulations were carried out in double precision using a commercial FE code (LS-DYNA, 2016) (version 9.3).

### 2.1 Subject-specific femur models

Since females after the age of 50 are more prone to femur fractures (Singer et al., 1998; Bergh et al., 2020; Kanis et al., 2005), in this study, female femurs with donor ages between 50 and 70 were used. The upper age boundary (70) was chosen to avoid femurs with reduced bone quality since previous studies revealed 2.7 times higher osteoporosis rate in patients above 70 compared to the age range of 50–70 (Kanis et al., 2013).

Subject-specific femur finite element models with heterogeneous material properties were generated using input CT scans. To achieve a comparable mesh in all femur models, a reference mesh was morphed into each of the femur geometries without adversely changing the mesh quality. Thereafter, local material properties were mapped based on CT greyscale values.

The full body female CT scans were accessed from the New Mexico Decedent Image Database (Edgar et al., 2020) along with the corresponding metadata summarizing the cadaveric anthropometry. The CT scan resolution was 1 mm × 1.025 mm with a slice thickness of 0.5 mm. The femur CT scans were selected excluding the decedents with a known history of muscular-skeletal diseases, diabetes, autoimmune diseases, long-term substance use, skeletal trauma, cadaveric decomposition, and presence of metal artifacts (LeBoff et al., 2022). Age, BMI, and stature statistics of the femurs (N:18), which met the selection criteria, were also analyzed to ensure that no outliers were included (Table 1).

**TABLE 1:** Femur data set characteristics.

	Min	Max	Mean	SD
Age	50	70	57.72	5.61
BMI	18.59	36.11	27.18	6.09
Stature	151.16	176.00	161.68	6.99

The input femur set was also initially analyzed in terms of the correlations between the femur morphology and subject anthropometry. Results were compared with the literature to ensure that the selected femurs reflect the population-relevant morphological variations due to the changing anthropometry expected when using a large cohort (Table 2).

**TABLE 2:** Morphological changes observed in femur data set due to the changing anthropometry compared to literature. Similar tendencies were observed in all cases.

Pair	Literature	Femur data set (N:18)
Femur Length (mm)/Stature (m) Menéndez Garmendia et al. (2018)	317.0 (N: 30) (p < 0.0001)	246.0 (p: 0.00017)
Caput-Collum-Diaphyseal (CCD) Angle (°)/Age (years) Fischer et al. (2020)	−0.158 (N:1,639) (p < 0.001)	−0.581 (p: 0.093)
Cortical Thickness (mm)/Age (years) Thompson (1980)	−0.05 (N:36) (p < 0.001)	−0.073 (p: 0.04)

The CT scans were retrieved as a collection of sub-scans of the different regions of the body in which the femurs were usually split between lower limb and torso scans. Therefore, sub-scans were merged and cropped to provide a single CT scan comprising the left femur. The merged scans were evaluated at the stitching plane by comparing the averaged Hounsfield unit (HU) values to ensure continuity across neighboring regions. In all merged CT scans, the maximum deviation between adjacent regions did not exceed 29.4 HU. This deviation was considered small (approximately 2% of the HU range observed in femurs) for the overall mechanical behavior. In addition, the fracture results of each individual femur were subsequently examined to confirm that no fracture initiated at the stitching plane.

Femurs were initially segmented automatically within a threshold range of 250–2060 HU. The resulting segments were then manually refined by isolating the largest island, closing gaps, and trimming irrelevant extensions. Inner cavities were removed using Boolean and island removal operations. Finally, 1- and 2-mm smoothing filters were applied, and the segments were compared with the scans to ensure consistency. The final femur segments, representing the femur surface, were later used as the reference surface for subject-specific femur models. All CT scans were processed using 3D-Slicer software (version 4.8.1) (Fedorov et al., 2012).

A reference FE mesh was developed, starting from the femur model provided in the Open Viva Human Body Model (Östh et al., 2017), using linear, selectively reduced fully integrated hexahedral elements (LS-DYNA Manual, 2023) with an average edge length of 1.25 mm. A mesh sensitivity study was carried out to identify the optimal element size (Supplementary Material S1).

The reference mesh was morphed into the reference femur surfaces (i.e., femur segments), from the segmented CT scans, in two steps. First, the reference mesh was morphed using Kriging interpolations in the PIPER software (Beillas et al., 2016), which ensured global geometrical fitting such as the femur size, femur neck angle, femur head size, and position. In the kriging interpolation, a total of 110 interpolation points were defined across the femur surface. These points were placed consistently across all femurs to capture key anatomical features and to ensure correspondence between specimens. Specifically, interpolation points were distributed around the circumference of 13 anatomical regions, for example, the femoral head and neck, greater and lesser trochanters, femoral shaft, and distal condyles. The list and the description of the reference interpolation points used in Kriging interpolations are provided in the Supplementary Material S2. Next, the “reference to target” morphing function in ANSA software (version 19.1.1) (ANSA, 2018) was used to improve geometrical matching, namely, the normal distance between the surface nodes of femur mesh and the femur segments. Later, the final mesh quality was improved by applying smoothing functionality in ANSA.

In order to increase the resolution of the material property distribution and local bending in the thin cortical regions on the femoral surface surrounding trabecular bone, two solid elements with a constant 0.5 mm thickness (1 mm in sum) were used. After offsetting the femur mesh by 1 mm in the negative direction along the surface normal, the solid elements were extruded in the positive direction using the surface elements which ensured constant element thickness in whole femoral surface. An efficient enhanced strain formulation (LS-DYNA Manual, 2023) was applied on the thin surface elements to avoid over stiff behavior due to the low thickness of the elements. The enhanced strain formulation was developed by Borrvall (2009) to reduce transverse shear locking in fully integrated solid elements with poor aspect ratio.

Morphed femurs were compared with segment geometry according to the maximum normal surface distance. Changes in element quality due to morphing were assessed based on the average element size and the aspect-ratios.

Bone density of each specimen were mapped on the morphed mesh with the BONEMAT (version 3.2) software (Taddei et al., 2007) using the femur-specific CT scans. Merged and cropped CT scans were calibrated to hydroxyapatite densities (mgHA) based on the muscle-adipose-air calibration strategy proposed by Eggermont et al. (2019). In detail, the HU-mgHa calibration of the subject-specific CT scans was achieved correlating (using linear functions) the median HU values of the corresponding areas with the reference mgHA values of −840, −80, and 30 (mg $/{cm}^{3}$ ) given for air, adipose, and muscle sections (Eggermont et al., 2019). The air, adipose, and muscle sections for calibration were generated covering 2 ${cm}^{2}$ areas in 3 neighboring slices. Later, the ash density ( $\rho_{ash}$ ), the apparent density ( $\rho_{app}$ ) and the elastic modulus were calculated according to the equations provided by Enns-Bray et al. (2018) based on Morgan, Bayraktar, and Keaveny (2003) and Schileo et al. (2008). The calculated elastic modulus values were used for both trabecular and cortical bone materials.

A density threshold of 1.4 (gr $/{cm}^{3}$ ) (Enns-Bray et al., 2018) was applied to separate cortical and trabecular bone regions. Additionally, thin solid elements on the outer surface of the femur were modeled using cortical bone material, regardless of their densities. Considering the differences in constitutive material behavior, in this study, trabecular and cortical bone were modeled separately to ensure realistic model behavior of the whole femur in all tested load cases. Cortical bone was modeled using a metal plasticity model (Mat_124) (Khor et al., 2018). Trabecular bone was represented using a crushable foam model (Mat_83) (Enns-Bray et al., 2018), which was particularly important to reflect large compressive deformations common under fall-induced proximal femur fractures.

In both bone materials, tension-compression asymmetry was applied using the nonlinear material curves presented by Enns-Bray et al. (2018) material (Sup. 3). For trabecular and cortical bone, the yield and ultimate stress values were defined separately based on BMD values as described by Enns-Bray et al. (2018). Additionally, the rate dependency proposed by Enns-Bray et al. (2018) was applied to the trabecular bone. For cortical bone, rate effects were neglected since, in the literature, there is no clear consensus regarding the rate dependent tensile properties of cortical bone (Hansen et al., 2008; McElhaney, 1966; Cronin et al., 2022). The used material curves are presented in the Supplementary Material S3 based on the parameters defined by Enns-Bray et al. (2018). Apart from the material curves, an element erosion criterion was defined such that the elements subjected to a first principal strain greater than 0.2 (Fleps et al., 2018) were deleted from the simulation.

The resulting subject-specific femur modelling strategy were verified in three-point bending, axial torsion, and lateral fall load cases (as described in Section 2.4 in greater detail) in terms of their ability to predict previously reported femoral fracture load variability due to the changing anthropometry, age, and areal bone density. For this purpose, model performance was evaluated qualitatively based on three femur models (Table 3). The selection of individual femurs aimed to cover a wide range in age and height, thereby providing a cohort comparable to the experiments as presented in Table 3. (Funk et al., 2004; Martens et al., 1980; Courtney et al., 1994). Subject-specific femur verification is discussed in detail in Supplementary Material S4.

**TABLE 3:** Anthropometric information of the verification models along with the reference experimental studies (3p: Three-Point, FN aBMD: Femur neck areal bone mineral density, sd: Standard Deviation).

	Age	Sex	Stature (cm)	BMI	FN aBMD (g/cm2)	Ref
Femur 1	45	F	174	19.6	0.84	-
Femur 2	37	M	179	25.4	0.86	-
Femur 3	77	F	156	17.5	0.53	-
Averages	53	-	169	20.8	0.74	-
3p Bending	59 (sd:10)	8M	177 (sd:10)	27 (sd:6.5)	-	Funk et al. (2004)
Axial Torsion	56 (sd:13.2)	33M/14F	-	-	-	Martens et al. (1980)
Lateral fall	52 (sd:10.1)	18 (M-F)	-	-	0.78 (sd: 0.25)	Courtney et al. (1994)

### 2.2 Principal component analysis for the density distribution and geometry

All femur models had an identical mesh regarding the node and element numbering and relative position of the nodes in terms of the anatomical attributes. However, due to the distinct position of each femur in space, models needed to be aligned with rigid body transformations based on the reference femur. This alignment enabled the analysis of the relative shape variations between the individual femur models. Alignments were carried out using a Python script which conducted a Procrustes Transformation (PT) (Langron and Collins, 1985). PT defines the rigid body transformation between the geometries with shape deviations such that the minimum average distance between the nodal coordinates is achieved. In the case of the femur models, the average distance was calculated between the surface nodes.

Next, geometries and material properties of the heterogeneous femur models were separately analyzed with principal component analysis (PCA) (Géron, 2019). Concerning the geometries, the PCA was conducted based on the nodal coordinates. In the case of the heterogeneous material properties, the element densities, which are the primary determinant of elastic modulus, yield-, and ultimate-stress values (Supplementary Material S3), were used to construct the PCA.

Accordingly, the PCA enables the representation of the geometry and density distribution of each femur based on principal component (PC) vectors and the corresponding principal values (PV). The following mathematical operations were applied within the PCA (Equation 1):

$$s_{i}={\left[x_{1},y_{1},z_{1},x_{2},y_{2},z_{2},\ldots ,x_{n},y_{n},z_{n}\right]}^{t},or\;s_{i}={\left[d_{1},d_{2},\ldots ,d_{n}\right]}^{t}$$ (1)

where $s_{i}$ are the element densities or the vectorized nodal coordinates of a femur (called by the subscript *i*) representing the density distribution (statistical appearance model) or geometry (statistical shape model) respectively (n stands for the number of nodes or elements).

The covariance matrix ***D*** was defined following a mean normalization ( $\left.ds_{i}\right)$ , where *N* stands for the number of femurs (Equation 2):

$$D=\frac{1}{N}\sum_{i=1}^{N}\left(ds_{i}\right){\left(ds_{i}\right)}^{t}:ds_{i}=s_{i}-s_{mean\;}\;\&\;s_{mean}=\frac{1}{N}\sum_{i=1}^{N}s_{i}$$ (2)

Singular value decomposition was applied on the covariance matrix where the columns of the left singular vector *U* represented the PCs (Equation 3):

$$D=UWU^{t}=\left[\begin{array}{ccc} \vdots & \vdots & \vdots \\ {pc}_{1} & \ldots \;{\;pc}_{j}\;\ldots & {pc}_{N}\\ \vdots & \vdots & \vdots \end{array}\right]WU^{t}:\left|{pc}_{j}\right|=3n\;|\;n$$ (3)

The PVs of the subject-specific femurs were determined as follows (Equation 4):

$${pv}_{i}=U^{t}ds_{i}$$ (4)

In order to quantify the contribution of each PC in the representation of the reference femur set, a compactness test was conducted (Bonaretti et al., 2014), where each reference femur was reconstructed using an increasing number of PCs. Results of the compactness tests were evaluated separately for geometry and density distribution in terms of the absolute mean errors. To achieve this, nodal coordinates and the element density values of the reconstructed femurs were compared with the reference femurs.

The relationships between the subject-specific PVs and the anthropometric variables, namely, the BMI, stature, and age were analyzed using linear regressions, where results are presented as a correlation matrix. In order to demonstrate the particular variations represented by individual PCs new femur models were generated using ±SD PV of the corresponding PC. Concerning the density distribution, the influence of the PCs was demonstrated without including the average, which reflected the change over the average density distribution due to the given PC (Equation 5). In terms of the geometry, PCs were included in the average shape such that a manipulated average femur shape was created (Equation 6).

$$s_{density\;variation}=+{SD}_{j}\;{pc}_{j}$$ (5)

$$s_{geometry\;variation\;}=s_{mean}\;\pm {SD}_{j}\;{pc}_{j}$$ (6)

### 2.3 Development of the parametric femur models using multilinear regressions

New femur instances for parametric models were generated using the PCs and corresponding contribution factors $b_{j}$ (Equation 7). The contribution factors ( $b_{j}$ ) were estimated using multilinear regressions, to capture the interdependencies where each input parameter influences all contribution factors. The multilinear regression models were developed using the principal values and the anthropometric parameters (BMI, Stature, Age) of the individual femurs to predict corresponding contribution factors for a given stature, BMI and age values. The multilinear regression models were trained in Python and in order to ensure that the parameter initiations did not influence the results, the regression models were trained multiple times using different libraries (Pedregosa et al., 2011). All training variations resulted in identical outcomes.

$$s_{new}=s_{mean}+\sum_{j=1}^{N}b_{j}\;{pc}_{j}:b=F_{regression}\left(Stature,BMI,Age\right)$$ (7)

The predicted contribution factors were used to generate parametric femur models (Equation 7), which capture the stature-, BMI-, and age-related variations in geometry and BMD distributions. Through the multilinear regressions, 13 femur models were generated varying one parameter at the time, referring to the input cohorts mean as well as ±1 and ±2 standard deviation (SD) stature, BMI and age values (Table 4). Using the ± second SD values, the min–max range of the input cohort was covered, except for the maximum age, which differed by 1.06 years. As mentioned previously, the input cohort was also analyzed initially to eliminate outliers (in terms of the patient anthropometry and femur morphology) and ensure the regression results were not biased due to the influence of individual samples.

**TABLE 4:** Anthropometric variations used in femur model generation, applying multilinear regressions.

	-2SD	-1SD	Mean	+1SD	+2SD
Stature	147.00	154.69	161.68	168.67	175.66
BMI	15.00	21.09	27.18	33.27	39.00
Age	46.50	52.11	57.72	63.33	68.94

### 2.4 Assessment of the redundancy of the developed parametric femur modeling methodology

To evaluate the redundancy of the developed parametric modeling methodology and the reference femur set, a leave-one-out analysis was performed. Parametric femur models originally generated for ±1SD variations (Table 4) using the entire data set were created again, leaving each time one of the reference femurs out. In other words, 7 parametric femurs (Average, ±1SD Stature, ±1SD Age, ±1SD BMI) were generated 18 times, leaving out one of the reference femurs (N:18) each time. The results of each test were compared with the generated parametric femurs using the complete training set in terms of the mean absolute error. Subsequently, the overall performance was evaluated separately for density distribution and geometry based on the maximum mean absolute error value encountered within all comparisons.

### 2.5 Assessment of the developed parametric femur modelling methodology in terms of the morphological changes and femoral fracture load variations

The developed parametric femur models were compared with the reference subject-specific femur set in terms of the morphological changes. For this purpose, CCD angles were defined based on the angle between the femur shaft and neck axis, and the cortical bone thickness was determined at the anterior mid-shaft region. Mid femur shaft cortical bone area was calculated in Primer Software (version 14) (Primer, 2018) using the “cut-section-area” functionality, only considering the cortical bone elements. The used definition provided the cross-section area of the selected elements on the defined cross-section plane which was placed at the mid-length of the femur and oriented perpendicular to the femoral axis. Results were compared in terms of the trendline slopes (linear regression functions) of stature-femur length, age-CCD angle, and age-cortical bone thickness.

In order to assess whether parametric femur models can predict the femoral fracture load variations associated with the changing anthropometry observed within the set of reference femurs, all femur models were tested in three-point bending, axial torsion, and lateral fall load cases.

**FIGURE 2:**
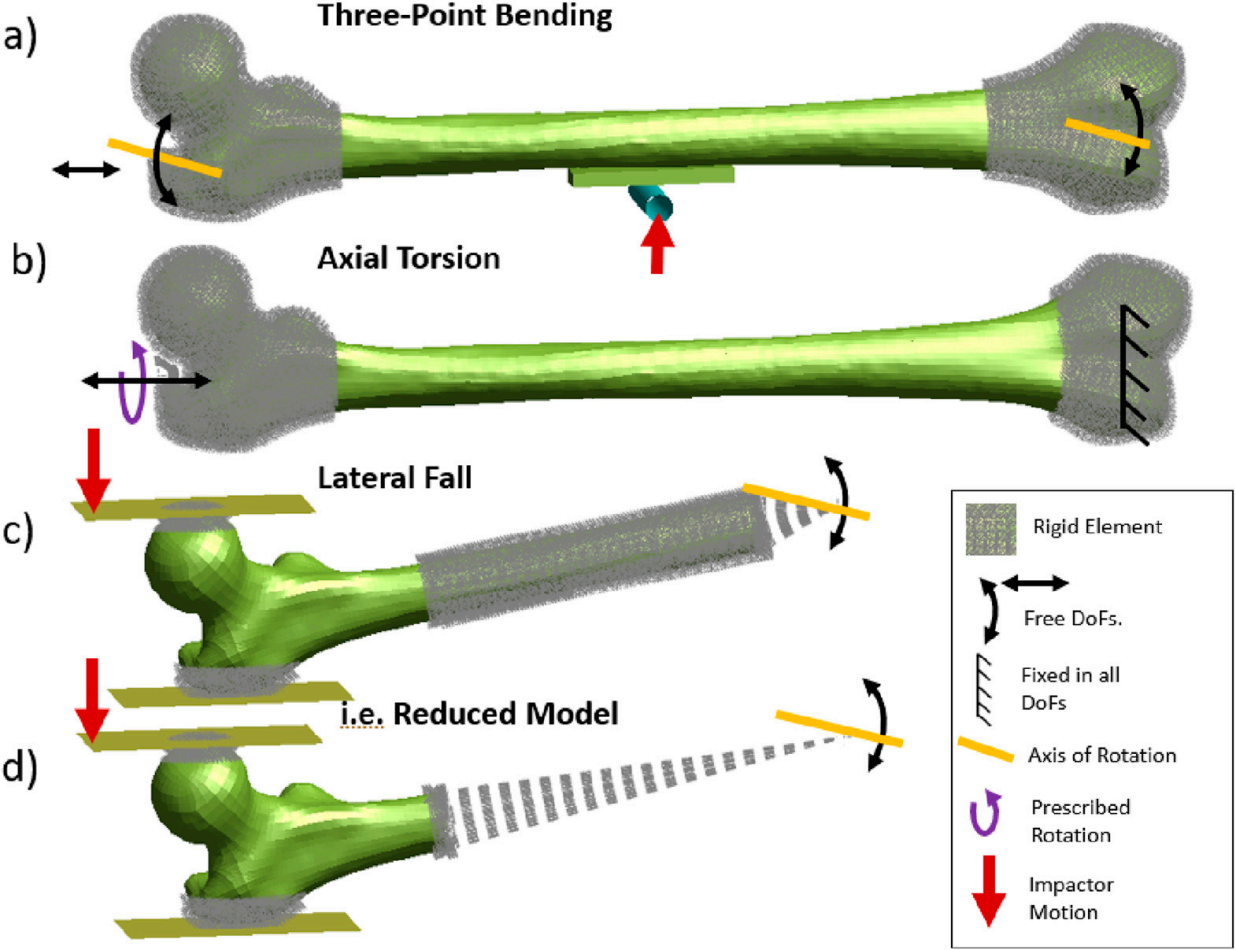
Boundary conditions of three-point bending **(a)**, axial torsion **(b)**, and lateral fall **(c)** loading. A reduced modeling strategy **(d)** was applied to assess femur fractures (DoF: Degrees of Freedom) in all load cases.

Femoral fracture load under bending loading was determined applying three-point bending load case according to the experiments published by Funk et al. (2004) (Figure 2a). According to the publication, the proximal and distal ends of the femurs (8 cm from the tip-point) were embedded (enclosed with a polymer, casted in a cylindrical metal pot). Both ends were allowed to rotate in the coronal plane, where the proximal end was allowed to move in the superior-inferior direction. A rigid cylinder-shaped impactor with a diameter of 12 mm was positioned in the middle of the proximal and distal center of rotations. The impactor accelerated for 10 ms with a ramp function to avoid initial coupling induced vibrations and later moved with a constant velocity of 1.2 m/s in the medial direction. The contact forces between the impactor and the femur were monitored along with the impactor displacement. Additionally, in order to eliminate contact force fluctuations and increase the accuracy of the fracture force measurements, a foam block, reported by Ivarsson et al. (2009), was positioned between the femur shaft and the impactor (Figure 2a).

Axial torsion was simulated based on the experimental study presented by Martens et al. (1980) (Figure 2b). The distal and proximal femur were embedded where the distal femur was constrained in all global translation and rotations, and the proximal femur was allowed only to rotate around and move in the superior-inferior axis. A prescribed rotation with a constant rotation rate of 0.7°/ms around the femoral axis was applied. The applied torque during the prescribed rotation was measured.

The proximal femoral fracture load under lateral fall loading was assessed according to the experimental study described by Courtney et al. (1994) (Figure 2c). Femur models were positioned with 10° adduction and 15° internal rotation with the distal femur embedded and constrained, only allowing rotations in coronal plane. The contact surface of the femur head and the greater trochanter were reinforced with rigid elements to avoid local crushing of the bone, to represent the spherical metal plates attached to bone in experiments. The femur head was deformed with a constant velocity of 100 mm/s using a rigid impactor where the contact forces between the impactor and the femur head were measured.

All three-point bending, axial torsion, and lateral fall simulations were conducted using reduced models, where the embedded sections were eliminated and represented with rigid elements to reduce the computation time (i.e., Figure 2d). The comparison provided within the subject-specific model verification (Supplementary Material S4) showed that the model reduction has no considerable influence on the results.

#### 2.5.1 Evaluation of predicted fracture load variations in terms of the weighted sum of differences

Femoral fracture load variations observed using parametric femur models, due to the changing stature, BMI, and age were compared with the results of reference subject-specific femur set. Results were evaluated according to the slope of the trendlines (based on the ultimate loading -stature, -BMI, -age pairs).

The overall error evaluation was designed to penalize strong correlations or large differences. It was achieved by assessing the error as the weighted sum of differences. This also enabled avoiding biased percentage difference values when dealing with small reference trend line slopes (divided by small values close to zero).

In order to quantify errors of the parametric femur models, regarding the differences observed in trendline slopes, initially a min-max normalization was applied based on the maximum and minimum values observed in the reference femur set (Equation 8). This normalization eliminated the unit differences between different output-input parameter combinations such as fracture force (kN)-stature (m), or fracture moment (Nmm)-Age (year), etc. Thereafter, subtracting the normalized slope values $\left(k_{yx}^{Norm}\right)$ , absolute differences $\left({Diff}_{yx}^{Norm}\right)$ between the reference femur set and the parametric models was calculated for each input-output pair (Equation 9) and maximum (reference or parametric) normalized slope values $\left(k_{yx\;\mathrm{Max}\;}^{Norm}\right)$ of the corresponding input-output combinations were calculated (Equation 10). Subsequently, weighted sum of differences ( WSD ) was determined (Equations 11, 12) based on the corresponding weight factors $\left(w_{yx}\right)$ , defined depending on the maximum normalized slope values. Finally, the percentage differences ( ${Diff}^{\%}$ ) were defined normalizing the WSD values within the range of minimum and maximum trendline slopes of the reference femur set (Equation 13).

$$k_{yx}^{Norm}=k_{yx}\times \frac{x_{ref}^{\max}-x_{ref}^{\min}}{y_{ref}^{\max}-y_{ref}^{\min}}:y=\left(k_{yx}\times x\right)+b$$ (8)

$${Diff}_{yx}^{Norm}=abs\left(k_{yx\;Referance}^{Norm}-k_{yx\;Parametric}^{Norm}\right)$$ (9)

$$k_{yx\;\mathrm{Max}\;}^{Norm}=\max\left(abs\left(k_{yx\;Referance\;}^{Norm}\right)or\;abs\left(\;k_{yx\;Parametric\;}^{Norm}\right)\right)$$ (10)

$$w_{yx}=\frac{k_{yx\;\mathrm{Max}\;}^{Norm}\;}{\sum k_{yx\;\mathrm{Max}\;}^{Norm}\;}:\sum w_{yx}=1.0$$ (11)

$$WSD=\sum {Diff}_{yx}^{Weighted}:{Diff}_{yx}^{Weighted}={Diff}_{yx}^{Norm}\times w_{yx}$$ (12)

$${Diff}^{\%}=\frac{WSD}{k_{Referance\;}^{Norm\;\max}-k_{Referance\;}^{Norm\;\min}}\times 100.0$$ (13)

The percentage differences were calculated for parametric models considering the morphological and femoral fracture load variations separately. Additionally, the statistical significance of the provided correlations and trendlines were evaluated in terms of the p-values and values above 0.01 considered to be non-significant (Li et al., 2023).

## 3 Results

### 3.1 Subject-specific femur models

Between the subject-specific femur models and original femur surfaces (generated by segmented CT scans), a maximum normal surface distance of 0.49 mm was observed in surface regions where fracture initiation was predicted (e.g., femur shaft or neck). In all models, the maximum normal surface distance was observed in the trochanteric fossa region (max. 4.1 mm). Due to the morphing operation, the average element size varied between 1.15 mm and 1.34 mm, where the average aspect ratio varies between 2.0 and 2.2.

**FIGURE 3:**
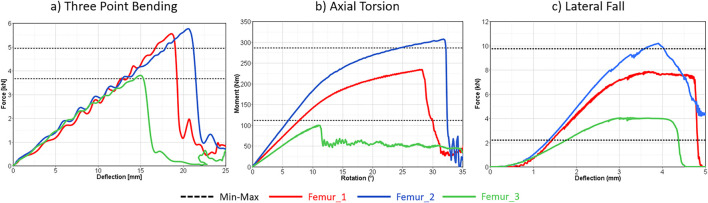
Subject specific femur model verification results in three-point bending **(a)**, axial torsion **(b)**, and lateral fall **(c)** loading. The dashed lines represent the min-max range of the corresponding experiments.

In terms of the reported fracture loading ranges, verification of the subject-specific femur modelling strategy showed the largest differences between femur 2 and the maximum 3p-bending fracture loading (0.7 kN), and femur 3 and the minimum lateral fall fracture loading (1.9 kN) (Figure 3). The average fracture loading of femur 1, 2, and 3 were %20, %16.05, and %16.20 higher than the reported average fracture loading in 3-point bending (Funk et al., 2004), axial torsion (Martens et al., 1980), and lateral fall (Courtney et al., 1994) experiments respectively. Additionally, all femurs showed comparable fracture patterns and locations similar to the experiments (Sup. 4).

Across all load cases, Femur 2 (a 37-year-old male, 179 cm) exhibited the highest fracture load, whereas Femur 3 (a 77-year-old female, 169 cm) yielded the lowest. Consistent with its younger age and larger size, Femur 2 showed increased fracture load compared to the maximum values reported in experiments (Table 3). In contrast, the axial torsion result of Femur 3 demonstrated reduced fracture load relative to experiments conducted on a cohort with an average age of 56 years. Verification results of the baseline femur modeling strategy are presented and discussed in detail in the Supplementary Material S4.

### 3.2 Principal component analysis for the geometry and density distribution

**FIGURE 4:**
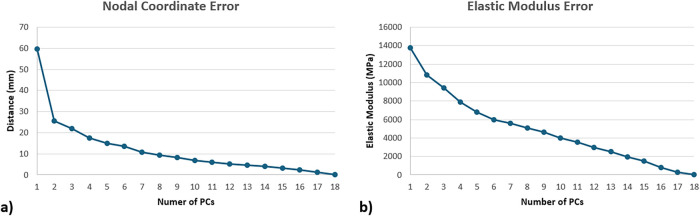
Compactness test results of the statistical shape **(a)** and appearance models **(b)**, represented in terms of mean absolute error, where femurs were recreated using increasing number of PCs.

Compactness test results showed an exponential decay in terms of the contribution of the increased number of PCs to the overall geometric representations (Figure 4a). Regarding the density distribution, a semi-constant decay was observed in terms of the contribution of the increasing number of PCs (Figure 4b).

**FIGURE 5:**
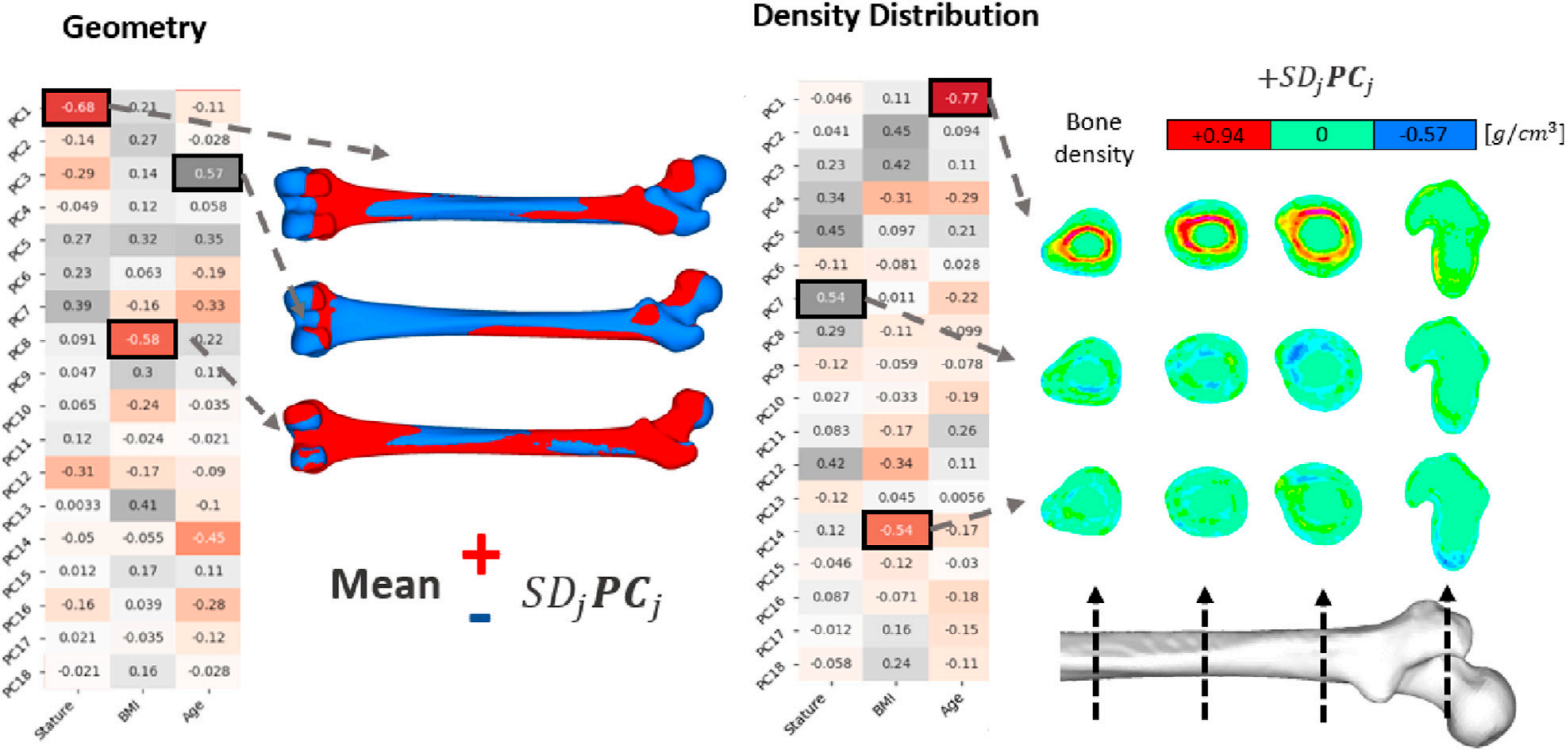
PCA results on femur geometry and density distribution. Correlation matrices show the Pearson correlation coefficients between the PVs (of the PCs) and the anthropometric inputs. Geometric influences of corresponding PCs were illustrated by adding (red) or removing (blue) PCs from the average shape (Equation 5). PCs related to density distribution were presented directly, exhibiting the density distribution changes governed by each PC.

Principal component analysis results were evaluated in terms of the linear correlations between the subject-specific PVs and the anthropometric parameters (Figure 5).

Regarding the density distributions, the highest Pearson correlation coefficient were observed with age-PC1 (R: −0.77), stature-PC7 (R: 0.54), and BMI-PC14 (R: −0.54) pairs (Figure 5). Results showed that PC1 governs cortical bone thickness. PC7 and PC14, on the other hand, exhibit directional changes in the density distribution on the femur shaft such that density decreases and increases locally. Density variations yield by these PCs were presented in Figure 5 in four cross sections at various locations from mid shaft to proximal region.

Concerning geometry, the highest Pearson correlation constants were determined for stature-PC1 (R: −0.68), BMI-PC8 (R: −0.58), age-PC3 (R: 0.57) pairs. PC1 and PC3 were related to variations in femur size and CCD angle, respectively. Similarly, PC8 was responsible for small variations in the greater trochanter and femur head form. These variations were visualized in Figure 5 by multiplying the PCs with one standard deviation of the corresponding PVs and adding (red) or removing (blue) them from the average femur geometry (Equation 6).

### 3.3 Verification of the developed parametric femur modelling methodology in terms of the morphological changes

Leave-one-out tests resulted in a maximum mean absolute nodal coordinate error of 1.54 mm. Regarding the density distribution the maximum mean absolute error was 0.15 g/cm3. The observed maximum nodal coordinate errors were distributed in either proximal or distal epiphysis. In the case of the density distribution the observed maximum errors were located on the endocortical boundary due to the large density gradient between bone marrow and cortical bone.

Based on the anthropometric input representing the average and ±1 and ±2 SD of the input cohort (Table 4), new femur models were created using the parametric models. Geometries and density distributions of these models are provided in the Supplementary Material S5. Parametric femur models exhibit increased femur length with increased stature, local shape variations with increased BMI, reduced femur neck angle and decreased cortical and trabecular bone mass with increased age. Additionally, increased BMI resulted in a slight decrease in cortical bone and a clear increase in trabecular bone volume.

The reference femur set was compared with the parametric femur modeling results in terms of morphological changes due to anthropometric variations and the average values. Influences of stature on the femur length (Fem. L. – Sta.), age on CCD angle (CCD Ang. – Age) and age on cortical bone thickness (Cort. T. – Age) were analyzed based on the min-max normalized slope of the corresponding trendlines (Table 5). Original trendline slope values (without normalization) are provided in Supplementary Material alongside the corresponding p-values (Supplementary Material S5).

**TABLE 5:** Comparison of reference femur set and the parametric femur model in terms morphological changes relative to the input variables (Stature, BMI, and age). Blue and orange show weight factor (Equation 11) and the weighted differences (Equation 12), respectively.

	Ref.	Param.	W.	W. Err
Fem. L. - Sta.	0.811	0.787	0.457	0.011
CCD Ang. - Age	-0.394	-0.514	0.29	0.035
Cort. T. - Age	-0.445	-0.449	0.253	0.001

Regarding morphological variations, the parametric femur model was compared with the reference femur set and found to have weighted sum of differences (WSD) (Equation 12) of 0.047. Additionally, average percentage difference on morphological variations between the reference and parametric femur model was calculated (Equation 13) as 3.719%.

The created average femur (Av.) showed good agreement with the average values of the reference femur set in terms of the femur length (Av.: 431.2 mm vs. Ref.: 432.3 mm) and mid femur shaft cortical bone area (Av.: 347.9 mm^2^ vs. Ref.: 355.2 mm^2^).

### 3.4 Verification of the developed parametric femur modelling methodology in terms of the femoral fracture load variations in 3p-bending, axial-torsion and lateral fall loading

**FIGURE 6:**
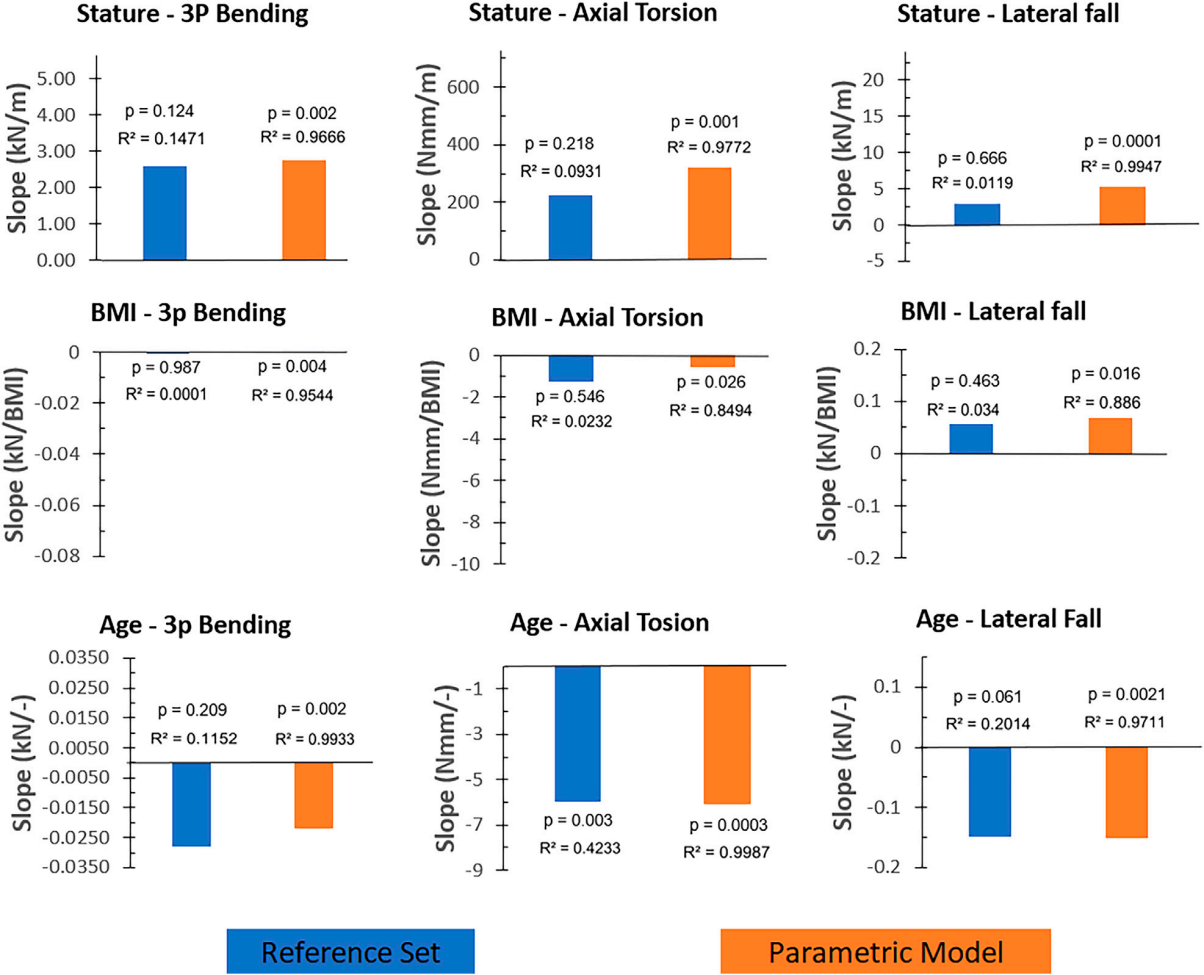
The trendline slopes (without normalization) of the reference femur set and the parametric femur results given for load cases (three-point bending, axial torsion, and lateral fall) and input parameters (stature, BMI, and age). Blue and orange color present reference femur sets and parametric femurs, respectively. Determination and p-values of the trendlines were also provided for comparison reasons. Axis scales were adjusted based on the min-max values of the corresponding scatter plots (Supplementary Material S5) to reflect the relative magnitude of the trendline slopes.

Simulation results of the reference femur set and the parametric femur models were evaluated using scatter plots of failure loading and the corresponding stature, BMI, or age inputs, provided in the Supplementary Material S5. Figure 6 shows the trendline slopes of the reference femur set and the parametric model where the determination coefficients are also provided to represent the scatteredness.

In all load cases, results showed comparable trendline slopes between the reference femur set and the parametric femur models (Figure 6). In the case of the three-point bending and axial torsion, age and stature yield correlations with fracture loading. In the case of the lateral falls, results showed that the fracture loading was mainly influenced by age. The only significant correlation observed using the reference femur set was in the case of the axial torsion and age (p: 0.003), where a 2.1% relative difference in trendline slope values was observed between the reference femur set and parametric femur models.

**FIGURE 7:**
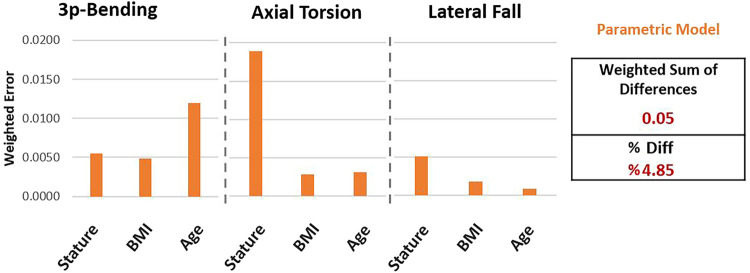
Differences observed using parametric femur models compared to reference femur set in terms of the weighted differences.

Weighted differences for parametric models compared to the reference femur set (Figure 7) showed larger values in three-point bending-age (0.012) and axial torsion-stature (0.018) cases as a result of the considerably large trendline slope differences, alongside the strong correlation observed in these cases (Figure 6). All other cases resulted in weighted difference values below 0.005 due to either negligible trendline slope differences or corresponding weak correlations.

The WSD of the parametric femurs was calculated (Equation 12) as 0.05. Accordingly, the percentage differences can be given (Equation 13) as 4.85% for the parametric model, normalizing the weighted sum of differences within the minimum and maximum normalized reference trendline slope range (−0.65, 0.50).

The average femur created using parametric modelling showed similar results with the reference femur set regarding the average femoral fracture load in three-point bending (Av.: 3.19 kN vs. Ref.: 3.28 kN) where the fracture occurred at the mid-femur shaft, in both femur sets. In lateral fall (Av.: 6.25 kN vs. Ref.: 5.79 kN) and axial torsion (Av.: 220 Nmm vs. Ref.: 202 Nmm) load cases, the femoral fracture loads were overpredicted by the average parametric femur with a factor of 7.9% and 8.9%, respectively.

## 4 Discussion

The aim of this study was to develop and test a novel parametric femur modeling methodology that can represent the influence of changing stature, BMI, and age in femoral fractures in three-point bending, axial torsion, and lateral fall cases, taking both geometry and density distribution of the whole femur into account. Results have shown that the developed parametric modeling strategy can predict the anthropometry-related variations in femoral fracture loading. The developed models can be used to investigate fracture risk associated with anthropometric variations in complex loading cases, where femoral loading cannot be reduced to an isolated region or a single loading mode.

So far, only a limited number of studies have incorporated parametric femur modeling in fracture assessments. This study is, to the best of our knowledge, the first to predict fractures across different load cases including both variations in density distribution and geometry of the whole femur. Furthermore, it is also the first parametric femur modeling study that investigates fracture load variations due to changing stature, BMI, and age to assess both femoral shaft and proximal femur fractures.

Unlike the previous parametric and statistical femur modeling studies, which aimed to make patient-specific predictions, this study aimed to develop parametric femur models to represent femoral fractures and fracture load variations based on anthropometric input. In other words, the developed parametric models can be used to understand the influence of the changing patient anthropometry on femoral fractures under various loading conditions. Considering that femoral loading also changes according to anthropometry, these models can be especially useful to develop strategies or devices to mitigate femur fractures for specific patient anthropometries.

Showing the relevance of the used input data, anthropometric changes and their relationships with the femur morphology in the subject-specific femur set were similar to those reported in the literature. Studies reported the influence of stature on femur length (Menéndez Garmendia et al., 2018), age on CCD angle (Fischer et al., 2020), and age on cortical bone thickness (Thompson, 1980). In all cases, the reference femur set exhibited similar results as reported in the literature, such as increased femur length with increasing stature, decreasing CCD angle and cortical bone thickness with increasing age.

Compactness test results yielded only a slight exponential decay in reconstruction error using increased number of PCs, which justifies the use of all PCs in the parametric models. Additionally, the leave-one-out test results showed only minor variations between the test results. This demonstrates that the parametric femur results presented in this study were not biased due to individual femurs.

Parametric models can capture most of the morphological variations due to the changing anthropometry within the reference femur set. Correlation results observed using the parametric model showed similar trends as the reference femur set. In terms of the morphological variations, parametric model resulted in percentage differences of 3.72% compared to the reference femur set.

Subject-specific finite element models of the reference femur set presented femoral fracture load variations due to the changing anthropometry similar to those reported in the literature, which emphasizes the relevance of the used input data in terms of the fracture load variations. The three-point bending results presented by Funk et al. (2004) showed that stature and BMI were positively correlated, and age was negatively correlated with the femur shaft fracture loading. Apart from the influence of the BMI, these observations were in line with the reference femur set results.

Concerning the lateral falls, Shen et al. (2015) reported increasing proximal femur fracture loading as a result of the increasing BMI. Similarly, the reference femur set also showed increased femoral fracture load with the increased BMI. In addition, a reduction in femoral fracture load with age was predicted in the reference femur set which is also in line with the results presented by Keaveny et al. (2009).

Calculated percentage differences of the parametric model (4.85%), compared to the reference femur set in terms of the fracture load variations due to the changing anthropometry, suggest that the developed parametric models can predict the fracture load variations of the reference femur set. Parametric model presented fracture load variations due to the changing stature, BMI, and age in all load cases similar to the reference femur set.

Fracture loading results of the developed parametric models showed clear linear dependency and strong correlations with the corresponding anthropometric input parameters. This can be mainly interpreted based on the eliminated scatteredness in terms of morphological changes achieved through the orthogonality of the principal components and the multilinear regression functions used, which eventually yield linear multifactorial dependencies between the input and output parameters. However, accordingly, the results also suggest a linear dependency between these morphological changes (as a result of the anthropometric variations) and the fracture loading response.

Concerning the evaluation of differences observed between the subject-specific reference femur set and the parametric femur model, applied quantification aimed to eliminate biases due to trendline slope values close to zero. Therefore, initially, a min-max normalization was conducted to eliminate unit differences. Later the overall difference was calculated in terms of the weighted sum of differences which ensured that the differences observed within the strong correlations (or large over-predictions of weak correlations) were penalized over the weak correlations. Percentage differences were provided using a second normalization based on the minimum and maximum normalized trendline values. The reason behind this quantification was to provide a value that evaluates the differences compared to the observed range of variations.

Previous studies showed femoral fracture load variations based on age and changing anthropometry using a large number of experiments or simulations (Funk et al., 2004; Shen et al., 2015; Keaveny et al., 2009). In the case of a different loading, a target cohort, or the presence of an orthopedic device, researchers still need to conduct a large number of experiments or simulations to investigate femoral fracture load variations similar to the previous studies. However, the presented novel parametric modeling methodology opens new possibilities to investigate femoral fracture load variations based on the changing stature, BMI, and age. Such models can allow to investigate femoral fracture load variations under different scenarios with reduced costs using only a few simulation models. For example, to understand the influence of changing the age in the presence of an orthopedic device, the average model, and its variations (±sd. age) created using parametric modeling can be investigated by applying the relevant loading conditions rather than testing or simulating different cohorts with a large number of samples.

None of the previous studies have developed a femur modeling strategy using heterogeneous material properties for the whole femur to represent femur shaft and proximal femur fractures. Therefore, in this study, apart from the developed parametric model, a consistent femur modeling strategy was introduced for the whole femur. The verification results (Sup. 4) showed that the subject-specific femur models could reflect the expected influence of changing stature, BMI, and age on the femoral fracture load variations. The verification results also showed that the implemented femur modeling strategy could capture the mechanics of the femur shaft and proximal femur fractures based on the fracture patterns and locations.

The main limitation of this study is the number of included samples. Using eighteen input femurs, it is not possible to generate representative models for the whole population. Therefore, this work remains a methodological study documenting the outcomes of the parametric modeling approach regarding its capabilities to predict the influence of changing anthropometry in femoral fractures.

Regarding the failure loading and the anthropometric input correlations observed in the reference femur set, only the result pairs of age-axial torsion results showed significant correlations. Due to the cohort differences between the previous studies (of femoral fracture load variations due to changing anthropometry) and reference femur set, rather than a quantitative evaluation, as reported earlier, results were compared in terms of the increasing or decreasing femoral fracture load values.

The material properties were defined using linear and exponential functions, where each material parameter can be expressed in terms of each other. In this study, density was chosen as the input parameter for the statistical appearance model, which defines the distribution of material properties. Hence, the average model was generated primarily by averaging the density values. However, due to the nonlinear relationship between bone density and material properties, this averaging caused a slightly different average mechanical behavior compared to the reference femur set. Accordingly, average failure loading was slightly overpredicted in axial-torsion by the average parametric model compared to the reference femur set.

Since subject-specific femur models were not validated against subject-specific experiments, one can argue that the validation status of the used subject-specific femurs poses a limitation. The developed femur modeling strategy was considered a tool to demonstrate the performance of the parametric modeling strategy. Results showed that the used subject-specific femur models can reflect the expected fracture force variations and fracture behavior due to the changing anthropometry. This was considered sufficient to test the performance of the developed parametric modeling strategy, which was the main aim of this study. The validity of the baseline models used in the parametric model was only considered relevant to the representability of specific population groups and the direct clinical applicability of the developed parametric model.

Whether the observed differences between the parametric model and reference set are acceptable depends on the biomechanical application. The level of acceptable difference should be decided based on the impact of the decision (Viceconti et al., 2021). From that perspective, for example, when the developed parametric model is used to determine the fixation strategy of femur shaft fractures based on the changing patient age concerning the torsional loading, introduced deviations (2.0%) can be considered acceptable. However, when the developed parametric model is used to optimize the positioning of a hip stem to reduce the periprosthetic shaft fracture risk under bending conditions based on the changing stature, the introduced deviation (9.9%) can be considered large since the success of a THA application are known to be highly sensitive to the positioning of the stem (Shishido et al., 2018).

A direct comparison between the reported values in this study and the previous studies was not possible due to the differences in modeling strategy and the considered load cases. To our knowledge, the most relevant study was published by Klein et al. (2017), where homogeneous femurs and calibrated material models were used, and models were tested in combined axial compression and 3-point bending loading.

Additionally, in this study, only the left femurs were selected as the reference; therefore, any morphological differences that might exist between the left and right sides, possibly due to the dominance of one side (Taddei et al., 2016), were not considered.

Further research is required to understand the fracture loading variations due to the changing anthropometry by focusing on different cohorts than those presented in this study. In future studies, male femurs, or increased cohort age could be analyzed further and compared with the results of this study in terms of the morphological or fracture loading variations (Dudle et al., 2024). Particularly, increased age would reveal valuable insights regarding osteoporotic bone and deviations in its fracture behavior as a result of the changing anthropometry, which is a major factor in fall-induced loading condition (Sarvi and Luo, 2019).

## 5 Summary

In order to investigate the influence of the changing patient anthropometry in femoral fractures, a large number of experiments or simulations are required. Parametric femur models offer an alternative to examining fracture risk for different patient anthropometries and correspondingly changing loading conditions with reduced cost. Such models are particularly useful in the development of orthopedic devices or fracture prevention solutions. However, no parametric femur models reported in the literature have so far addressed femoral fractures under various loading conditions, considering both the geometry and density distribution of the whole femur. Accordingly, the aim of this work was to develop and evaluate a novel parametric femur model that uses stature, BMI, and age as input parameters, while accounting for both the geometry and density distribution of the entire femur, to predict fracture loads under lateral fall, axial torsion, and shaft bending conditions.

For this purpose, a subject-specific femur modeling strategy that can represent proximal and diaphyseal femur fractures was introduced and verified based on the available literature. Later, parametric femur models were developed, combining the principal component analysis with multilinear regression results. Assessment results showed that the developed parametric femur model could predict the proximal and diaphyseal femoral fracture load variations associated with changing stature, BMI, and age of a reference femur set.

In the future, similar models can be used in orthopedic device development or *in silico* trials for patient-group-specific fracture assessments to study the influence of the changing anthropometry in different load cases without conducting a large number of subject-specific simulations.

## Data Availability

The raw data and the datasets supporting the findings presented in this study will be provided for scientific use by the authors upon reasonable request. Requests to access the datasets should be directed to sara.checa@tuhh.de.
