# Supplementary material for: Parametrized statistical appearance and shape modelling strategy to predict proximal and diaphyseal femoral fractures
Source: Front Bioeng Biotechnol. 2025 Nov 3;13:1693678. doi: 10.3389/fbioe.2025.1693678 (PMC12620422; doi:10.3389/fbioe.2025.1693678)
Supplement: Supplementary file 1 [file Supplementaryfile5.pdf]

Supplementary Material 5

Extended Results

Pair	Literature	Ref. Femur Set (N:18)	Parametric Model (N:5)
**Femur Length (mm) / Stature (m)**<br><br>(Menéndez Garmendia et al., 2018)	317.0 (N: 30) (p<0.0001)	246.0 (p: 0.00017)	239.3
**CCD Angle (°) / Age (years)**<br><br>(Fischer et al., 2020)	-0.158 (N:1639) (p<0.001)	-0.581 (p: 0.093)	-0.759
**Cortical Thickness (mm) / Age (years)**<br><br>(Thompson, 1980)	-0.05 (N:36) (p<0.001)	-0.073 (p: 0.04)	-0.0736

Table S5-1: The reference femur set and the parametric femur models were compared to literature in terms of the correlations between the femur morphology and anthropometry.

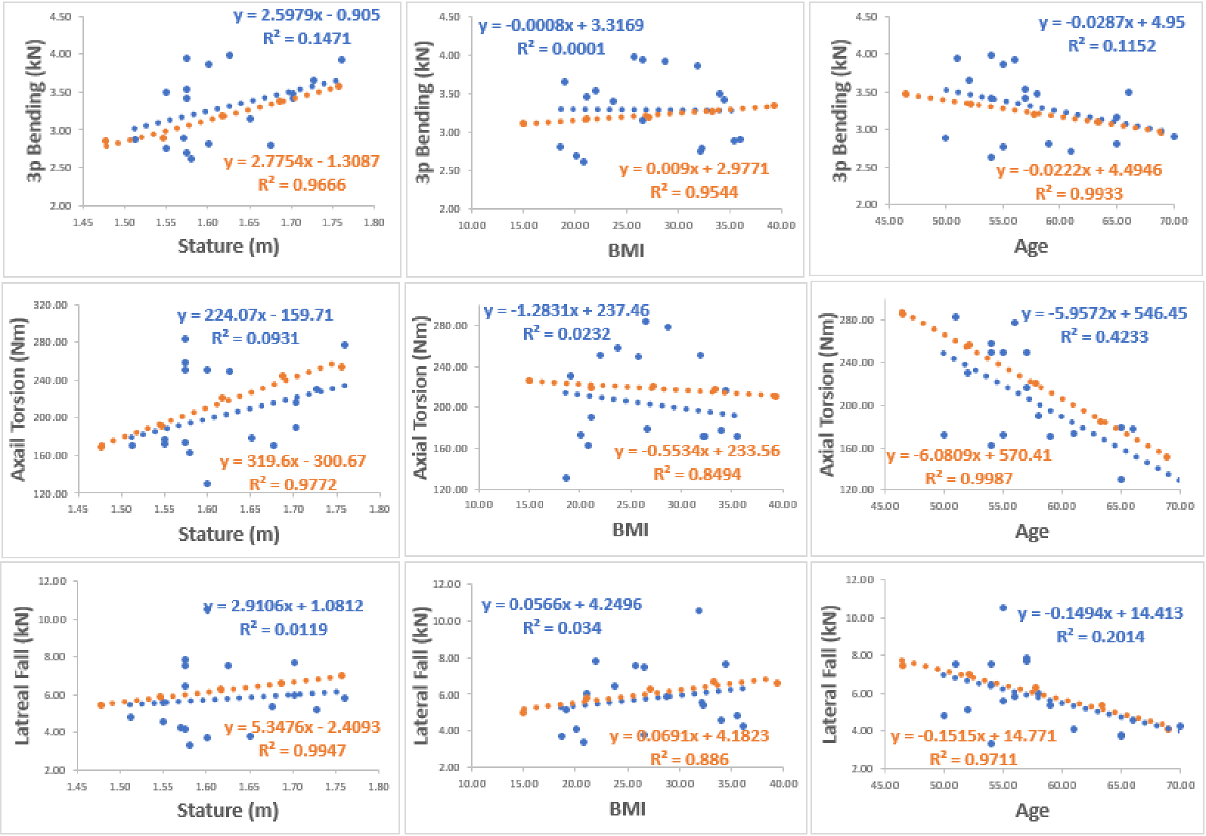

Figure S5-1: Scatter plots show failure loading (in three-point bending, axial torsion, and lateral fall cases) and input parameter (stature, BMI, and age). Trendlines of the scatterplots describes the failure load variations based on the input parameters. Blue and orange colors present reference femur set and parametric femurs, respectively. Determination coefficients of reference femur results were also provided for comparison reasons.

	Stature (m)	BMI	Age	3p-Bending (kN)	Axial torsion (Nm)	Lateral fall (kN)	Femur lenght (mm)	Cortical thickness (mm)	CCD Angle°
max-min	0.25	17.52	20.00	1.36	181.18	7.20	75.54	3.28	29.51

Table S5-2: Min-Max ranges used for the normalization.

Min-Max Normalized

	Stature				BMI				Age			
	Ref.	M-Lin.	W.	W. Err	Ref.	M-Lin.	W.	W. Err	Ref.	M-Lin.	W.	W. Err
3p-Bend.	0.5059	0.5059	0.1665	**0.0000**	-0.0103	0.1159	0.0382	**0.0048**	-0.4118	-0.3235	0.1355	**0.0120**
Ax.-Tors.	0.3072	0.4382	0.1442	**0.0189**	-0.1238	-0.0535	0.0407	**0.0029**	-0.6568	-0.6712	0.2209	**0.0032**
Lat.-Fall	0.1004	0.1845	0.0607	**0.0051**	0.1363	0.1679	0.0553	**0.0017**	-0.4139	-0.4194	0.1380	**0.0008**

Parametric Model

Weighted Sum of Differences

0.05

% Diff

% 4.24

Table S5-3: The normalized trendline slopes (Fig 4.) of the reference femur set (Ref.) and the multilinear (M-Lin.) parametric femur results, where W. and W.err stands for weight factors and weighted difference values. Blue and the red color tones emphasis the magnitude of weight factor and the weighted differences.
