# Supplementary material for: Parametrized statistical appearance and shape modelling strategy to predict proximal and diaphyseal femoral fractures
Source: Front Bioeng Biotechnol. 2025 Nov 3;13:1693678. doi: 10.3389/fbioe.2025.1693678 (PMC12620422; doi:10.3389/fbioe.2025.1693678)
Supplement: Supplementary file 2 [file Supplementaryfile1.pdf]

# Supplementary Material 1

## Mesh Sensitivity

The mesh sensitivity analysis was carried out based on a 58-year-old female femur. The femur was modeled as it was described in Section 2. Mesh refinement was realized by splitting the hexahedral elements into eight elements. As a result, three femur models with an average element length of 2.5, 1.25, and 0.615 mm were generated. In all models, the material properties were mapped as was given in Section2. Mesh sensitivity was tested in three-point bending and lateral fall load cases following the reduced modeling strategy (Sec. 2). Different than the boundary conditions described in Section 2, in the lateral fall load case femur heads were loaded in force driven fashion such that the force was applied using a step function (1kN/ms).

The mesh sensitivity analysis results showed similar fracture patterns in all load cases for all refinement levels (Fig. S1-1). The sensitivity analysis results were evaluated concerning the fracture loads and displacements (Table S1-1).

| | Average Size | Fracture Load | Fracture Displacement |
|---|---|---|---|
| Three-point bending | 2.5 mm | 5.14 kN | 14.3 mm |
| | 1.25 mm | 5.74 kN | 13.2 mm |
| | 0.615 mm | 5.73 kN | 13.1 mm |
| Lateral Fall | 2.5 mm | 7.5 kN | 7.6 mm |
| | 1.25 mm | 6.6 kN | 5.8 mm |
| | 0.615 mm | 6.0 kN | 4.0 mm |

Table S1-1: Mesh sensitivity analysis results

Three-point bending results showed convergence with the mesh refinement both in terms of the fracture load and displacement. The contact force fluctuations observed in the 1.25 mm average element size resulted from the element erosions on the contact surface of the impactor. Since primary fracture initiation was always on the opposite side of the femur and the last surface element erosion took place 2.5 ms before the primary fracture, it was assumed that the previous surface element erosions under the impactor did not influence the fracture force and displacement results.

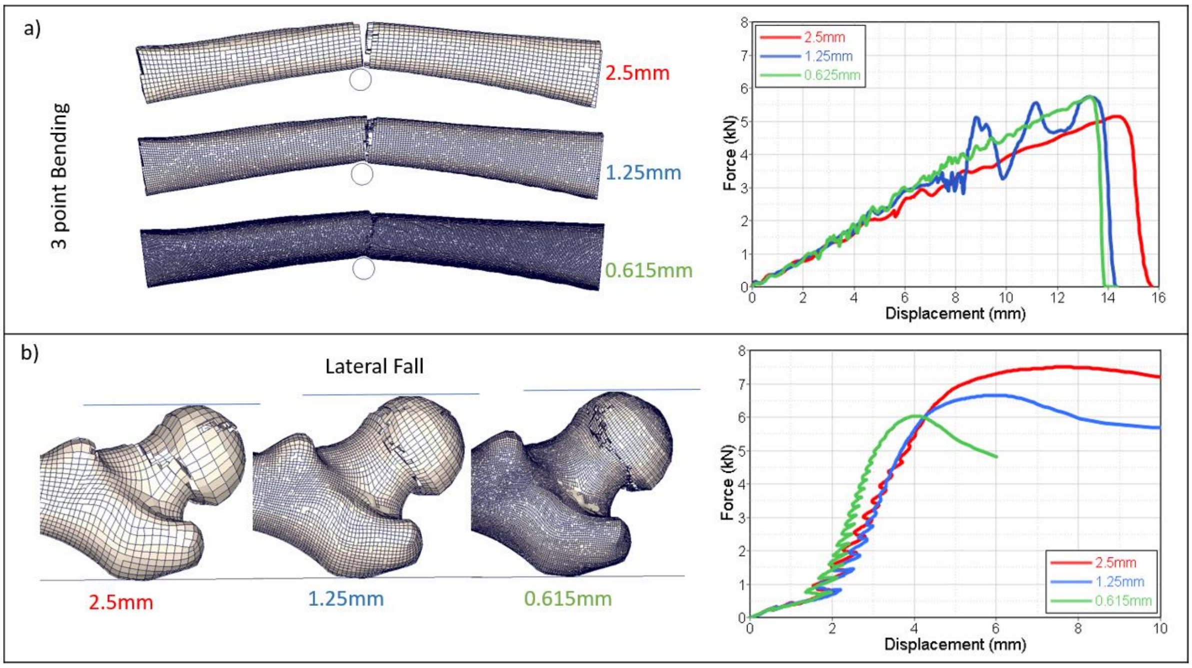


Figure S1-1: Mesh sensitivity analysis results.

On the other hand, in case of the lateral falls, although the fracture force difference between models decreased with mesh refinement, indicating that results started to converge, a full convergence was not reached with the simulated refinement level. Based on the convergence observed in three-point bending where the material distribution resolution did not change due to the element size as a result of the semi-homogenous material properties, it can be suggested that the slow convergence in the lateral fall case is related to the increased heterogeneity of the proximal femur. The refined models can capture the material properties with higher resolution, and therefore they can represent the mechanical properties more realistically. Such as, the increased initial stiffness with mesh refinement, which was observed in both load cases, can be explained with the more accurate representation of the cortical boundaries (especially in the femur head). It can also be suggested that any further mesh refinement providing an element size smaller than the source CT scan should not influence the mechanical properties.

Since a FE simulation with further refinement could not be computed due to the hardware limitations, mesh sensitivity was further investigated based on the accurate representation of heterogeneous material properties. Therefore, a further refined model was generated by splitting the elements of the model with an average element size of 0.615mm. Keeping in mind that the fractures under lateral loading were observed in the femur head and neck, material properties were only evaluated in these regions. Due to the stretched elements in head and neck regions, the average element size was noted as 3.9, 1.95, 0.975, 0.4875 mm (Fig. S1-2).

Given the pixel size of the reference CT scan (1.0 x 1.02 x 0.5 mm) (fig. S1-2), it can be suggested that the finest model can capture the maximum resolutions that the CT scan can deliver. Therefore, the finest model was considered as the reference to investigate how accurately the heterogeneous material properties were represented with a courser mesh. For this purpose, the elastic modulus spectrums of the models were analyzed using normalized histograms and the corresponding distribution plots (Fig. S1-3a). Later the elastic modulus distribution of each model was compared with

the reference model in terms of the Pearson correlation coefficients to quantify the similarity between the heterogeneous material property distributions.

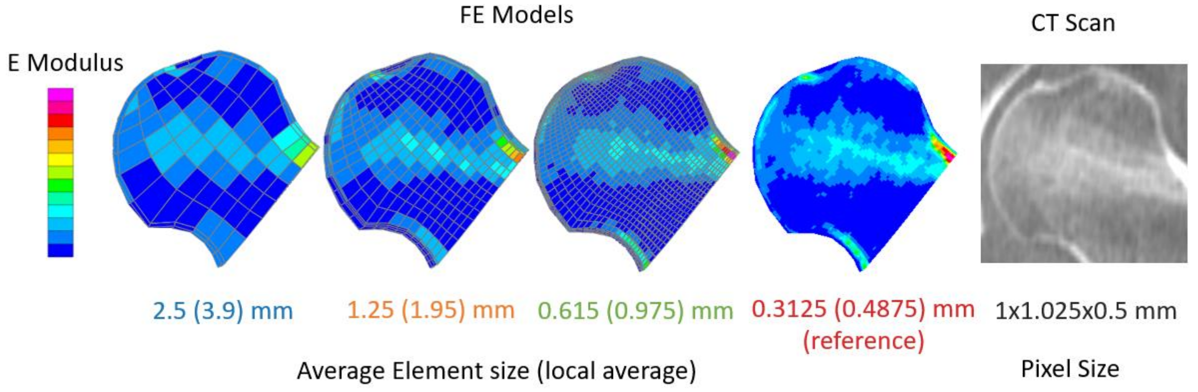


Figure S1-2 Increasing mesh density and resolution of the heterogeneous material properties compared with the source CT scan.

Results showed converging correlation coefficients with a 0.001 discrepancy between the reference model and the model with an average element size of 0.615 mm (fig. S1-3b). Considering this, it can be suggested that the FE simulations should also show converging results between these two models regarding fracture loading. Therefore, suggesting that the model reaches convergence in lateral fall load case with an average element size of 0.615 mm, the 1.25 mm average element size poses a 10% difference in fracture load.

Considering the convergence observed in three-point bending, the above suggested 10% difference in lateral fall cases, and the computational burden of a further refined models, the average element size of 1.25 mm was selected as the optimum mesh refinement level for this study.

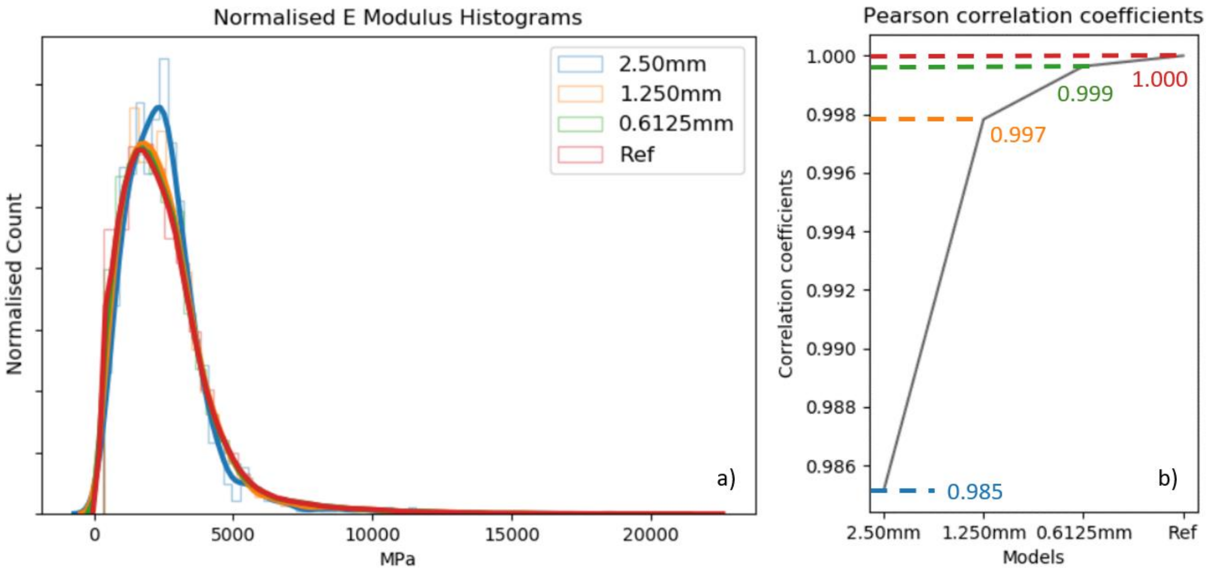


Figure S1-3 a): Normalized elastic modulus histograms and distribution curves. b): Comparison of the distribution curves with the reference model
