# Supplementary material for: Parametrized statistical appearance and shape modelling strategy to predict proximal and diaphyseal femoral fractures
Source: Front Bioeng Biotechnol. 2025 Nov 3;13:1693678. doi: 10.3389/fbioe.2025.1693678 (PMC12620422; doi:10.3389/fbioe.2025.1693678)
Supplement: Supplementary file 3 [file Supplementaryfile2.pdf]

Supplementary Material 2: **Target points in Kriging interpolation**

Reference points used in the Kriging interpolations are described in table S2-1.

No:	Description		No:	Description	
1	Femur neck axis intersection with the femur head segment	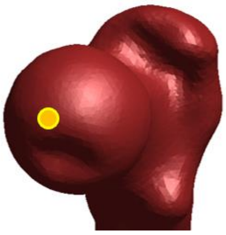	2-9	8 points on the subcapital femur head boundary (1,2)	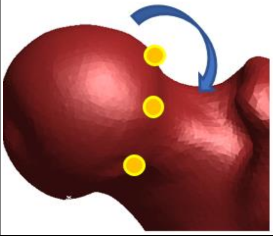
10-25	16 points between the point 1 and points 2-9 (1,2)	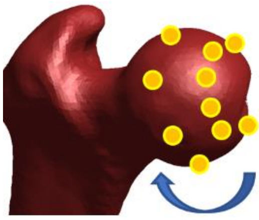	26-33	8 points on the subcapital femur neck boundary (1,2)	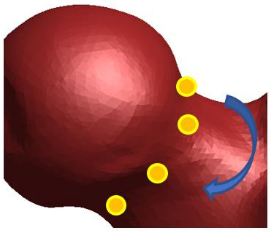
34-41	8 points on the mid cervical plane intersection (1,2)	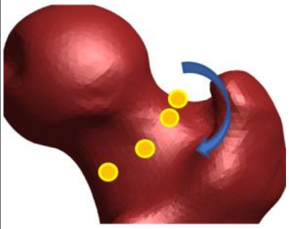	42-49	8 points on the transvers plane positioned 3 mm distal from the proximal tip of the greater trochanter (1,3)	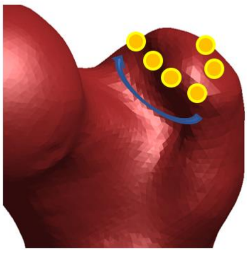
50-57	8 points on the plane defined between the anterior, posterior, and lateral tips of the greater trochanter	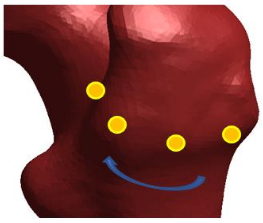	58-61	Tip of the lesser trochanter and 4 points, defined with 8 mm distance from the tip in proximal, distal, lateral, and medial directions.	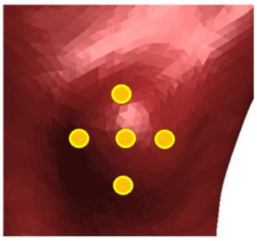
62-85	8 points defined on 3 transverse cross-section planes, positioned in the center of proximal and distal femur tips as well as a quarter femur length from the mid-plane in proximal and distal directions. (1,3)	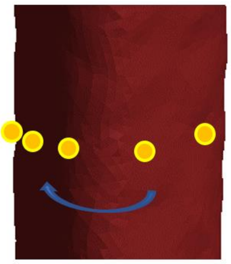	86-94	9 Points on: Posterior, anterior and distal tips of medial and lateral condyle; lateral and medial tips of lateral and medial epicondyle; and intercondylar fossa	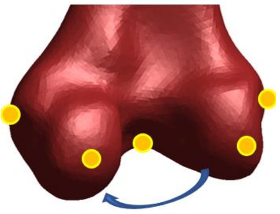
95-110	16 points defined on coronal planes positioned 10% the distance of posterior-anterior size of the distal femur from the posterior tips of the medial and lateral condyle	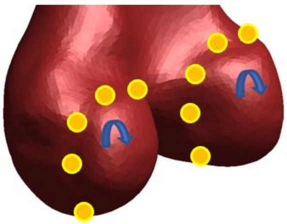	**Remarks:**<br><br>**1)** Projected counterclockwise with equal angles from the geometrical center of the cross-section line<br><br>**2)** Defined starting from the proximal intersection point of the femur segment and the proximal femur coronal plane<br><br>**3)** Defined starting from the medial tip of the femur segment and the corresponding plane intersection		

Table S2-1: Interpolation points used in femur morphing
