# Supplementary material for: Parametrized statistical appearance and shape modelling strategy to predict proximal and diaphyseal femoral fractures
Source: Front Bioeng Biotechnol. 2025 Nov 3;13:1693678. doi: 10.3389/fbioe.2025.1693678 (PMC12620422; doi:10.3389/fbioe.2025.1693678)
Supplement: Supplementary file 4 [file Supplementaryfile4.pdf]

Supplementary Material 4

Subject-specific femur modelling verification under three modes of loading

In order to verify the modelling strategy, performance of three subject-specific models was compared against the experimental literature in three different loading scenarios: three-point bending, axial torsion, and lateral fall load cases as described in the main manuscript. Femurs were selected to cover a large anthropometric variation and corresponding influences in the femoral fracture load. The average age, stature, BMI, and femur neck aBMD values of the selected femurs were within the first standard deviation of the reference experiments (Table 3).

Verification results were evaluated quantitatively for the average failure loading differences between the simulations and the experiments. Additionally, the femoral fracture load variations due to the changing anthropometry and age were qualitatively evaluated, comparing the subject-specific femur results with the experimental result corridors considering the age stature and BMI of the subject-specific femurs and the reference experiment. For example, in all load cases, the younger and larger femurs were expected to provide higher femoral fracture load than the smaller and older femurs, and based on the same principle, the subject-specific femur results were expected to fall within the right range compared with the experiments.

In three-point bending load case the contact forces between the impactor and the femur were monitored along with the impactor displacement and compared with the experimental results (Funk et al., 2004). Likewise, in axial torsion the applied torque during the prescribed rotation was measured and compared with the experimental results (Martens et al., 1980). The proximal femur fracture load was assessed according to the experimental study given by Courtney et al. (1994). Since the authors provided proximal femur fracture load results with respect to the femur neck areal BMD (FN aBMD) values, results were compared in terms of proximal femur fracture load vs. FN aBMD.

Areal bone mineral density (aBMD) measurements of the subject-specific femurs were calculated based on the description provided by the International Atomic Energy Agency (IAEA, 2010), dividing the hydroxyapatite (HA) mass of the region of interests (ROI) in FE models by the projection area of the ROI on the proximal femur coronal plane (Zhang et al., 2020) (Fig. S4-1).

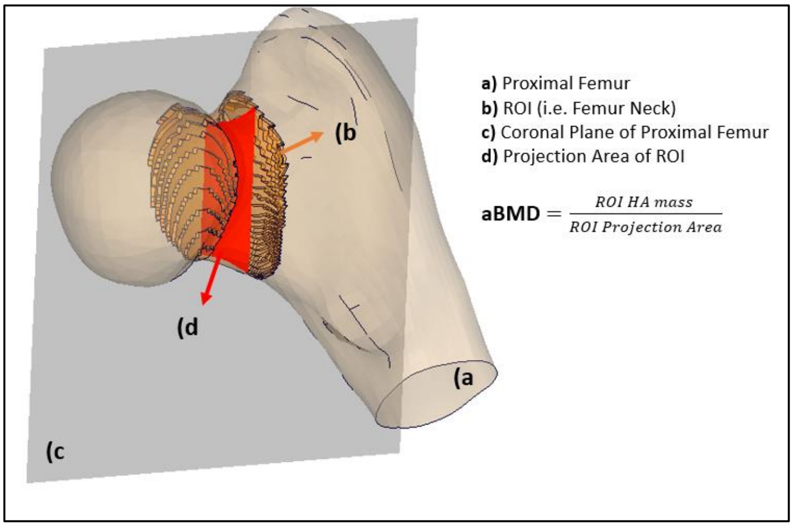

Figure S4-1: Areal Bone Mineral Density Calculations

In all load cases the embedded portions of distal or proximal femurs were modeled constraining the surface nodes of the corresponding femur segments using rigid elements. Additionally, in all load cases result of a reduced femur model, in which the embedded parts of the femur were erased and

represented only using rigid elements, was also compared with the original model (Femur1). The reduced models were used to decrease the computation cost of the parametric femur model assessments presented in main manuscript.

Subject-specific femur modelling verification results

Under three-point bending load, femurs 1 and 2 resulted in 0.9 and 1.3 kN greater fracture forces than the maximum value reported by Funk et al. (2004) (4.9kN), respectively (Fig. S4-3). On the other hand, Femur 3 showed a failure load within the first standard deviation of the experimental results (Fig. S4-3). All simulation results were in the given experimental deflection range (13.0-23.3mm) (Fig. S4-3). In all simulations, the fracture lines were aligned with the impactor and fractures were initialized on the medial side of the femur shaft. The reduced three-point bending model of femur1 provided identical results to the original model regarding the fracture load and deflection. Average failure load results of the simulations yield 21.01% difference in comparison to the experiments in three-point bending (table S4-1).

3 Point Bending

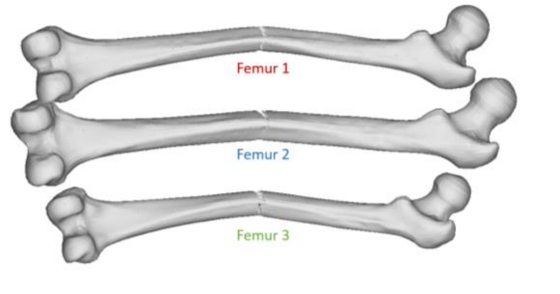

**F_fracture (kN)**	5.6	6.3	3.7
**Deflection (mm)**	20.0	21.4	15.5

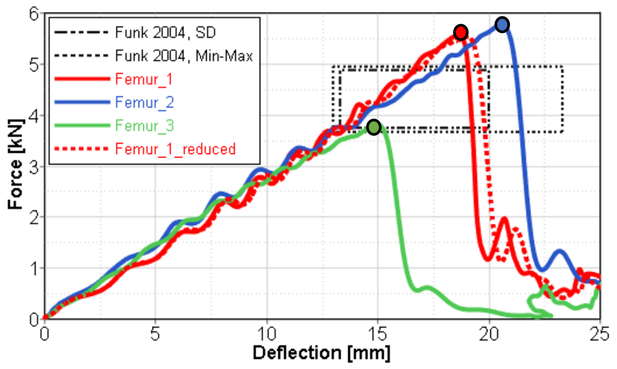

Figure S4-3 Three-point bending: Fracture patterns, failure load, and deformations, force-deflection curves along with the experimental result corridors.

All axial torsion simulations resulted in spiral fracture patterns (Fig. S4-4); however, the spiral fractures were not completely circumferential. In all models, the spiral fracture patterns were combined with fracture lines in the lateral-medial direction. Femur 3 provided a slightly lower fracture torque (100 Nm) and slightly higher fracture rotation (11.7°) than the experimental minimums reported by Martens et al. (1980) (111 Nm and 9.4°). On the contrary, Femur 2 resulted in higher fracture torque and rotation (306.8 Nm, 31.7°) than the maximum experimental values (286 Nm, 30.7°). The fracture torque and rotation results of Femur 1 were lower than the maximum experimental values. The reduced model performance was identical with the original model concerning the fracture torque, where only a -0.2° difference in the ultimate rotation was observed. Average failure loading results of the simulations yielded 16.70% difference in comparison to the experimental results in axial torsion (table S4-1).

The lateral fall simulation results were compared with the experimental results presented by Courtney et al., (1994) based on the fracture load and femur neck areal bone mineral density (FN aBMD) values. Fracture load and FN aBMD results of Femur 1 and 3 showed a similar gradient as the experimental linear regression function (11.17 vs. 10.87) with a bias of 0.69kN (Fig. S4-5). Femur 2 showed an increased failure loading (10.1 kN) than the experimental results for the corresponding FN aBMD value (0.84 g/cm2). The fracture load of Femur 2 (10.1 kN) also slightly exceeds the maximum femoral fracture load (9.75kN) reported by Courtney et al., (1994). Simulations showed femur neck fracture,

greater trochanter collapse, and sub-capital femur head fracture for Femur1, Femur2, and Femur3, respectively. In Femur2 following fracture initialization, an instant force drop was observed wherein in Femur 3 a force plateau was noticed during the crack propagation (Fig. S4-5). Observed average failure loading of the simulations was 16.05% greater than the experimental average in lateral falls (table S4-1).

Axial Torsion

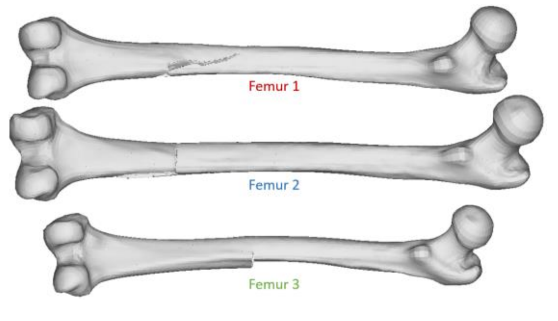

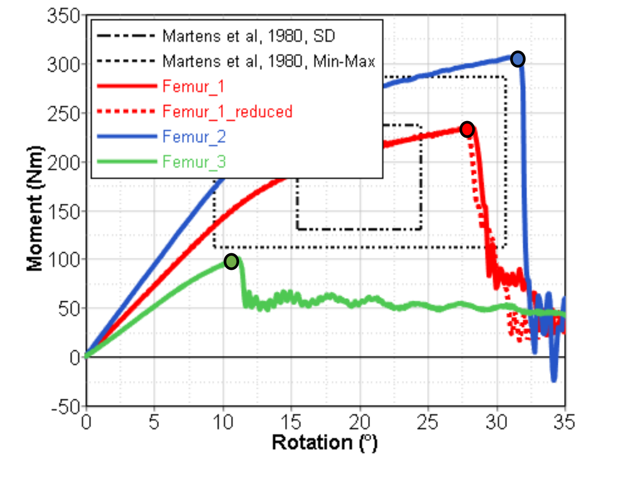

**T_fracture (Nm)**	234.1	306.8	100.2
**Rotation (°)**	28.2	31.7	11.7

Figure S4-4 Axial torsion results: Fracture patters, failure loading and deformations, torque-rotation curves along with the experimental result corridors.

Lateral Falls

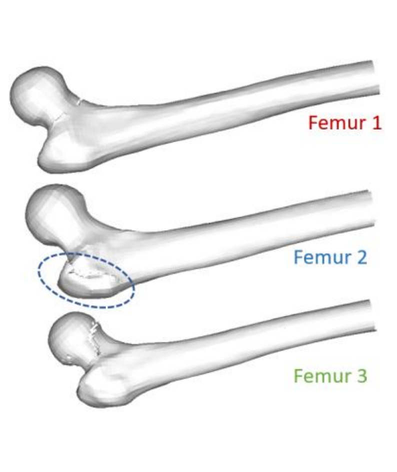

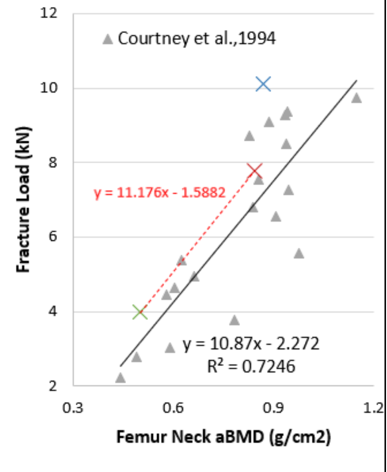

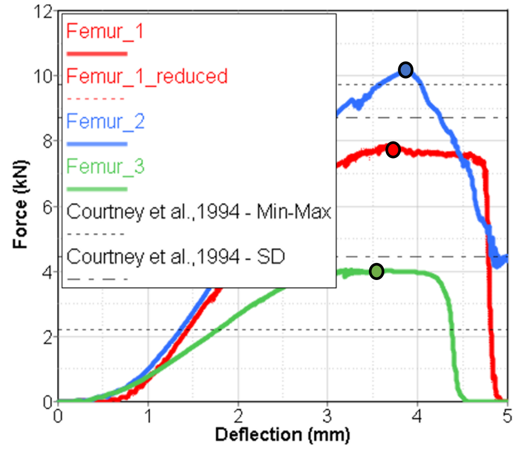

**F_fracture (kN)**	7.8	10.1	4.0
**Deflection (mm)**	3.6	3.8	3.0

Figure S4-5 Lateral Fall Results: Fracture patters, failure loading vs. femur neck areal bone mineral density, force-deflection curves along with the experimental result corridors.

	Experiments	Simulations	Difference
**3p-Bending**	4.2kN	5.2kN	21.10%
**Torsion**	183 Nm	213 Nm	16.70%

**Lateral Fall**	6.29 kN	7.3 kN	16.05%

Table S4-1: Average failure loading difference between the simulations and the experimental results

Discussion for subject-specific femur modelling verification:

A preliminary verification was conducted against the experimental data from literature using three subject-specific femurs to ensure models can represent femoral fractures realistically in three-point bending, axial torsion, and lateral fall load cases. The main aim of the verification was to ensure the used subject-specific femur modeling strategy could capture the influence of the anthropometric variations on the femoral fracture load. For this reason, a qualitative evaluation of the subject-specific femurs was conducted based on the provided result corridors and the available donor information for the experiments, such as age, stature, and areal bone mineral density (Table 3). Since the patient-specific CT scans were not available the verification results were evaluated quantitatively only in terms of the average failure loading results (Table S4-1).

In three-point bending, all femur models were in the stature range given by Funk et al. (2004). Considering the average age of the experimental cohort (59, sd: 10), it can be suggested that the fracture force results of the Femur 1 (37 years old) and Femur 2, which is greater than the reported experimental maximum, are plausible. On the other hand, the fracture force result of the Femur 3 was close to the reported experimental minimum, which is again reasonable according to the donor's advanced age.

The axial torsion experiment resented by Martens et al. (1980) reported only the age range of the donors (56, sd: 13.2) along with the mechanical results. Therefore, the simulation results are compared with the experimental result corridors based on the donor age of the subject-specific femur. Femur 2 (37 years old) resulted in higher fracture torque and rotation than the experimental maximum, where the Femur 3 (77 years old) showed lower fracture torque and slightly higher rotation than the experimental minimums. Femur-1 (45 years old) resulted in, on the other hand, fracture torque and rotation both was within the reported range. Taking the above-summarized model performances and the donor ages under consideration, the model behavior is considered plausible in axial torsion.

The lateral fall results were evaluated based on the fracture load and femur neck areal bone mineral density values provided by Courtney et al., (1994). Femur 1 and Femur 3 showed a close comparison with the experimental data regarding the FN aBMD – fracture load dependency. Femur 2, on the other hand, provided an increased fracture load for the given FN aBMD value. However, proximal femur fracture load depends on various factors like bone size, morphology, cortical bone thickness, and the distribution of the trabecular bone structures. For this reason, the FN aBMD alone is a weak indicator for proximal femur fracture load. Bearing in mind, Femur 2 belongs to a young male with above-average stature (37 years old, 179cm), and it showed a fracture load close to the experimental maximum (+0.35kN), the model behavior of Femur 2 can be considered plausible.

Based on the average failure loading results (Table S4-1) and the above-mentioned qualitative evaluation of the subject-specific femurs, it can be suggested that the followed modeling strategy can capture the mechanics behind the proximal femur and femur shaft fractures realistically. Accordingly, models provided the expected femoral fracture load variations associated with the changing anthropometry and age. However, due to the limited number of femurs used, it can only be considered as a preliminary verification. A validation of a novel femur modeling strategy that can represent femur shaft and proximal femur fractures, including rate effects and material asymmetry, goes beyond the primary motivation of this study.
