# Supplementary material for: Parametrized statistical appearance and shape modelling strategy to predict proximal and diaphyseal femoral fractures
Source: Front Bioeng Biotechnol. 2025 Nov 3;13:1693678. doi: 10.3389/fbioe.2025.1693678 (PMC12620422; doi:10.3389/fbioe.2025.1693678)
Supplement: Supplementary file 5 [file Supplementaryfile3.pdf]

Supplementary Material 3

Trabecular and cortical bone material curves

Figure S3-1 shows some examples of the trabecular and cortical material curves for various apparent bone mineral density values and strain rates (only in trabecular bone). The stress-strain pairs given in Figure S3-1 are explained in Table S3-1.

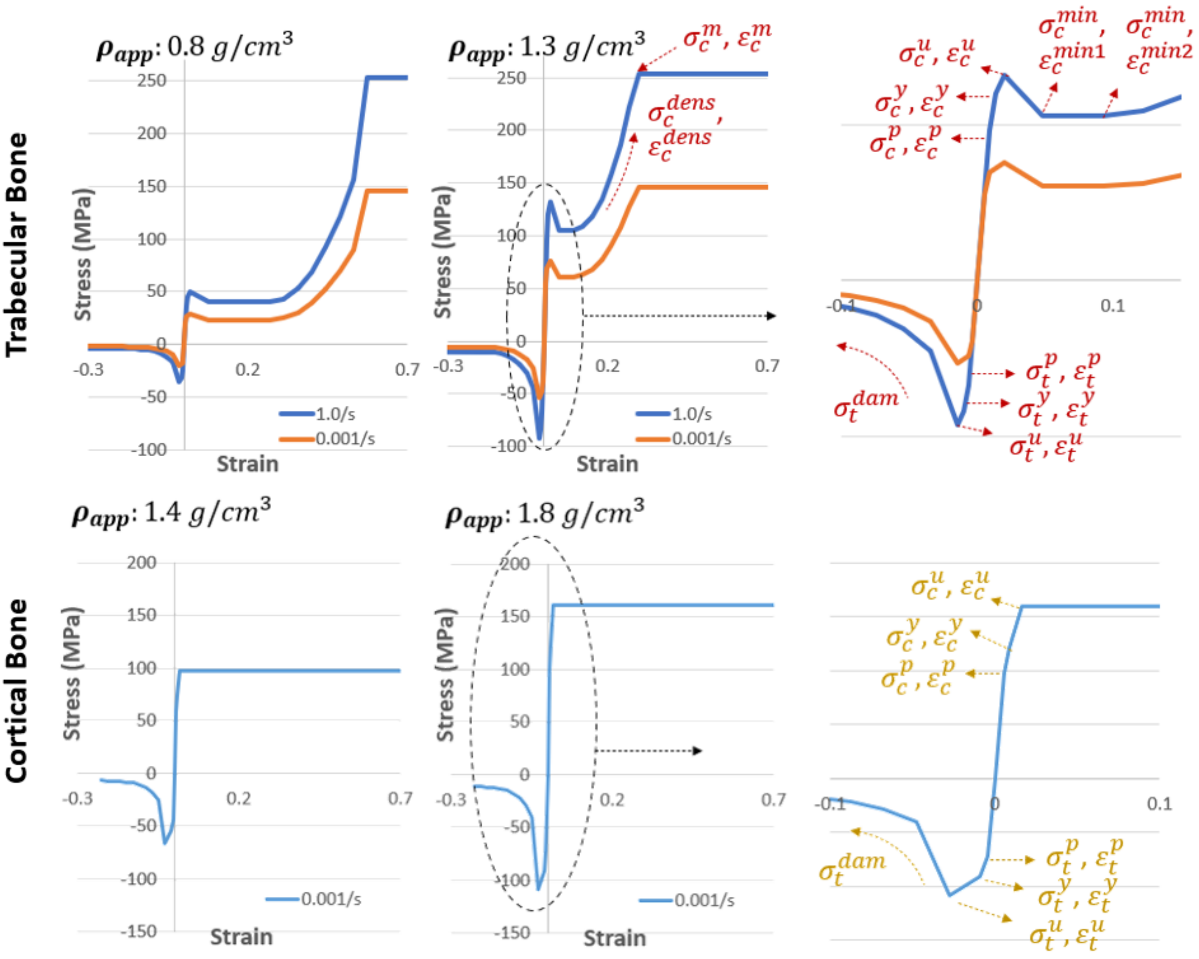

Figure S3-1: Trabecular and cortical bone material curves and the corresponding stress-strain pairs, defined based on the Ensy-Bray et al. (2018). ρ_{app}: Apparent density

	Parameter	Definition	Depends on	Defined based on	
Trabecular Bone			Tension		
	σ_t^p	Proportionality limit	σ_t^y	(Ariza et al., 2015)	Stress
	σ_t^y	Yield Stress	σ_t^u	(Helgason et al., 2008)	
	σ_t^u	Ultimate Stress	$\dot{\varepsilon}$, ρ_{app}, T/C	(Carter and Hayes, 1977)<br>(Bayraktar and Keaveny, 2004)	
	σ_t^{dam}	Post failure Damage	$\sigma_t^u, \varepsilon_t$	(Enns-Bray et al., 2018)	

	ε_t^p	Proportionality limit	σ_t^p , E	(Enns-Bray et al., 2018)	Strain
	ε_t^y	Yield Strain	σ_c^y , E, T/C	(Enns-Bray et al., 2018)	Strain
	ε_t^u	Ultimate Strain	ε_c^u , T/C	(Ariza et al., 2015)	Strain
	**Compression**				
	σ_c^p	Proportionality limit	σ_c^y	(Ariza et al., 2015)	Stress
	σ_c^y	Yield Stress	σ_c^u	(Helgason et al., 2008)	Stress
	σ_c^u	Ultimate Stress	$\dot{\varepsilon}$, ρ_{app}	(Carter and Hayes, 1977)<br>(Bayraktar and Keaveny, 2004)	Stress
	σ_c^{min}	Softening Stress	ε_c^u, ρ_{app}	(Helgason et al., 2014)	Stress
	σ_c^{dens}	Densification Stress	σ_c^{min} , σ_c^m , ε_c^{min2} , ε_c^m , ε_c	(Helgason et al., 2014)	Stress
	σ_c^m	Maximum Stress	$\dot{\varepsilon}$	(Helgason et al., 2014)	Stress
	ε_c^p	Proportionality limit	σ_c^p , E	(Enns-Bray et al., 2018)	Strain
	ε_c^y	Yield Strain	σ_c^y , E, T/C	(Enns-Bray et al., 2018)	Strain
	ε_c^u	Ultimate Strain	0.02	(Ariza et al., 2015)	Strain
	ε_c^{min1}	Softening Strain	ε_c^u, ρ_{app}	(Helgason et al., 2014)	Strain
	ε_c^{min2}	Pre-densification Strain	ε_c^u, ε_c^{min1}, ρ_{app}	(Helgason et al., 2014)	Strain
	ε_c^m	Maximum Strain	ε_c^u, ρ_{app}	(Helgason et al., 2014)	Strain

	Parameter	Definition	Depends on	Reference	
Cortical Bone	**Tension**				
Cortical Bone	σ_t^p	Proportionality limit	σ_t^y	(Ariza et al., 2015)	Stress
Cortical Bone	σ_t^y	Yield Stress	$\dot{\varepsilon}: 0.001/s$, ρ_{app} , T/C	(Carter and Hayes, 1977)<br>(Hansen et al., 2008)	Stress
Cortical Bone	σ_t^u	Ultimate Stress	$\dot{\varepsilon}: 0.001/s$, ρ_{app} , T/C	(Carter and Hayes, 1977)<br>(Hansen et al., 2008)	Stress
Cortical Bone	σ_t^{dam}	Post failure Damage	σ_t^u , ε_t	(Enns-Bray et al., 2018)	Stress
Cortical Bone	ε_t^p	Proportionality limit	σ_t^p , E	(Enns-Bray et al., 2018)	Strain
Cortical Bone	ε_t^y	Yield Strain	$\dot{\varepsilon}: 0.001/s$	(Hansen et al., 2008)	Strain
Cortical Bone	ε_t^u	Ultimate Strain	$\dot{\varepsilon}: 0.001/s$	(Hansen et al., 2008)	Strain

		Compression			
	σ_c^p	Proportionality limit	σ_c^y	(Ariza et al., 2015)	**Stress**
	σ_c^y	Yield Stress	$\dot{\varepsilon}: 0.001/s$, ρ_{app}	(Carter and Hayes, 1977)<br>(Hansen et al., 2008)	
	σ_c^u	Ultimate Stress	$\dot{\varepsilon}: 0.001/s$, ρ_{app}	(Carter and Hayes, 1977)<br>(Hansen et al., 2008)	
	ε_c^p	Proportionality limit	σ_c^p, E	(Enns-Bray et al., 2018)	**Strain**
	ε_c^y	Yield Strain	$\dot{\varepsilon}: 0.001/s$	(Hansen et al., 2008)	
	ε_c^u	Ultimate Strain	$\dot{\varepsilon}: 0.001/s$	(Hansen et al., 2008)	

Table S3-1: Summary of the stress-strain pairs, provided by Ensy-Bray et. al (2018), used in material curve definitions. E: Elastic Modulus (MPa), $\dot{\varepsilon}$: Strain rate, $\dot{\varepsilon}: 0.001/s$: Strain rate kept constant to eliminate rate effects in cortical bone parameters, ρ_{app}: Apparent density (g/cm^3), T/C: Tension-compression asymmetry scaling constant given as 0.70 and 0.68 for trabecular and cortical bones, respectively.
