# Supplementary material for: Parametrized statistical appearance and shape modelling strategy to predict proximal and diaphyseal femoral fractures
Source: Front Bioeng Biotechnol. 2025 Nov 3;13:1693678. doi: 10.3389/fbioe.2025.1693678 (PMC12620422; doi:10.3389/fbioe.2025.1693678)
Supplement: Supplementary file 6 [file Supplementaryfile6.pdf]

Supplementary Material 6

Generated parametric femurs based on the anthropometric input given in table 2

Below given image (Fig: S6-1) shows the geometrical differences as well as the variations in the elastic modulus spectrum of the elements between the average and femurs generated including +2sd stature, BMI, and age values using parametric femur models.

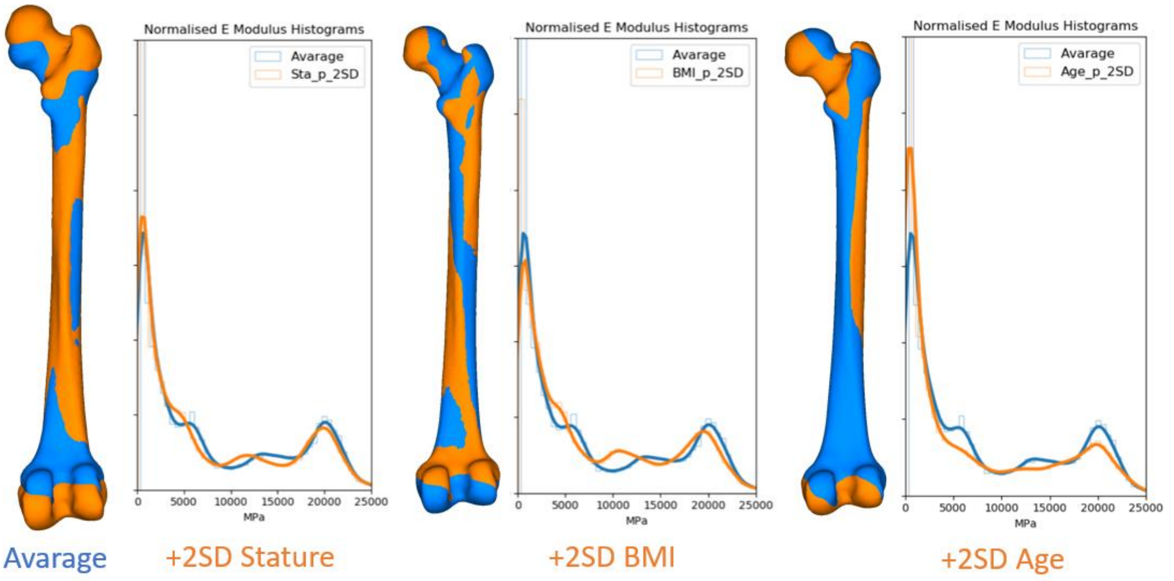

Figure S6-1: Generated femurs based on the age, BMI, and stature inputs (+2sd) using multi linear regressions.

Results show that parametric femur model exhibit increased femur length with increased stature, local shape variations with increased BMI, reduced femur neck angle and decreased cortical bone mass with increased age.

Elastic modulus spectrums (counted elastic modulus values of elements) describe the observed variations concerning the distribution of material properties. The most distinct peaks in spectrums represent the bone marrow and the cortical bone. Therefore, analyzing the elastic modulus spectrums (Fig: S6-1), an apparent reduction in cortical and trabecular bone mass can be identified due to the increasing age. Additionally, increased BMI resulted in slight decrease in cortical bone and a clear increase in trabecular bone volume.
